# TDDFT versus *GW*/BSE Methods for Prediction of Light Absorption and Emission in a TADF Emitter

**DOI:** 10.1021/acs.jpca.2c06403

**Published:** 2022-12-14

**Authors:** D. Chaudhuri, C. H. Patterson

**Affiliations:** School of Physics, Trinity College Dublin, Dublin 2, Ireland

## Abstract

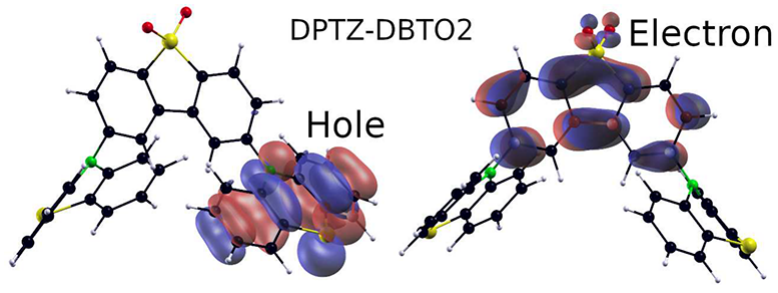

Design concepts for organic light emitting diode (OLED) emitters, which exhibit thermally activated delayed fluorescence (TADF) and thereby achieve quantum yields exceeding 25%, depend on singlet–triplet splitting energies of order *kT* to allow reverse intersystem crossing at ambient temperatures. Simulation methods for these systems must be able to treat relatively large organic molecules, as well as predict their excited state energies, transition energies, singlet–triplet splittings, and absorption and emission cross sections with reasonable accuracy, in order to prove useful in the design process. Here we compare predictions of TDDFT with M06-2X and ωB97X-D exchange-correlation functionals and a *G*_*o*_*W*_*o*_@HF/BSE method for these quantities in the well-studied DPTZ-DBTO2 TADF emitter molecule. Geometry optimization is performed for ground state (GS) and lowest donor–acceptor charge transfer (CT) state for each functional. Optical absorption and emission cross sections and energies are calculated at these geometries. Relaxation energies are on the order of 0.5 eV, and the importance of obtaining excited state equilibrium geometries in predicting delayed fluorescence is demonstrated. There are clear trends in predictions of *G*_*o*_*W*_*o*_@HF/BSE, and TDDFT/ωB97X-D and M06-2X methods in which the former method favors local exciton (LE) states while the latter favors DA CT states and ωB97X-D makes intermediate predictions. *G*_*o*_*W*_*o*_@HF/BSE suffers from triplet instability for LE states but not CT states relevant for TADF. Shifts in HOMO and LUMO levels on adding a conductor-like polarizable continuum model dielectric background are used to estimate changes in excitation energies on going from the gas phase to a solvated molecule.

## I Introduction

Simulation of TADF systems^1^ is commonly performed using time-dependent density functional theory (TDDFT) using density functionals that include a significant proportion of exact exchange.^2−6^ Mostly, vertical optical absorption spectra are calculated for molecules in their gas phase ground state equilibrium geometries and experiments are interpreted in light of results of those calculations. TDDFT has the advantage of being a relatively inexpensive method, allowing large organic systems to be treated using limited basis sets. The *GW* method,^7,8^ in which the electron self-energy, Σ, is evaluated to first order in the screened Coulomb interaction, *W*, and the one-electron Green function, *G*, is the state-of-the-art approach for calculating quasiparticle energies in bulk solids, surfaces, and nanostructures from first principles. These methods have also been used to calculate electron affinities (EA) and ionization potentials, (IP) of organic molecules^9−13^ and a combination of *GW* and Bethe–Salpeter equation (BSE) methods has been used to calculate excited states in organic systems.^14−16^ Both *GW* and BSE methods depend on screening of the electron–hole interaction to determine particle and hole energies and electron–hole excitations. Several groups have focused on the importance of mean-field starting point in a *GW* calculation and effects of self-consistency in *G* and *W* on predicted IP and EA values and excitation energies in molecules.^9,10,13,17^

**Figure 1:**
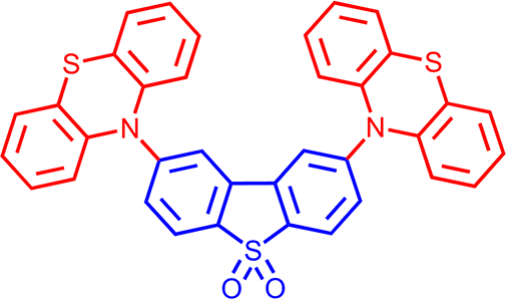
Molecular structure of the DPTZ-DBTO2 TADF emitter molecule. PTZ donors are red and the DBTO2 acceptor is blue.

The DAD system, 2,8-phenothiazine dibenzothiophene-*S*,*S*-dioxide (DPTZ-DBTO2) (Figure 1) is one of the most studied TADF emitter molecules, both by experiment^18−21^ and by simulation.^4,22−24^ It has also found applications such as in bioimaging and sensing.^25^

DAD TADF emitter molecules such as DPTZ-DBTO2 are designed to have small singlet–triplet splittings by ensuring that their lowest singlet states have strong donor–acceptor charge transfer (CT) character. This system and its DA counterpart, PTZ-DBTO2, have been extensively studied^4,22−24^ using the M06-2X functional.^26^ Conclusions drawn from that work depend on the specific ordering of low lying excited states of both local exciton (LE) and CT and singlet and triplet character. Here we compare TDDFT predictions of the excited states from the M06-2X functional to a range-separated functional, ωB97X-D,^27,28^ and *G*_*o*_*W*_*o*_@HF/BSE calculations. It is found that overall, both TDDFT/M06-2X and TDDFT/ωB97X-D yield similar predictions for excited state energies and absorption and fluorescence spectra, while *G*_*o*_*W*_*o*_@HF/BSE predicts a different ordering of excited state energies. *G*_*o*_*W*_*o*_@HF/BSE also suffers from the well-known triplet instability problem in predicting triplet excited state energies of LE states but not of CT states where electron and hole are well separated in space.

The M06-2X density functional performs well in benchmark studies of density functionals for TDDFT of both singlet and triplet states of organic molecules.^29−31^ DAD TADF systems exhibit both local exciton (LE) and charge transfer (CT) excited states, in which an excited electron and hole are both located on the D or A (LE) or are separated on D and A (CT). Methods for treating TADF systems must be able to treat short and long-range electron–hole interactions accurately in order to predict the relative ordering of LE and CT excited states. While the M06-2X functional performs well in benchmark studies of LE states in relatively small organic molecules,^29−31^ it contains a fraction of the Coulombic electron–hole attraction which is independent of the electron–hole separation. In contrast, the BSE method incorporates a screened electron–hole attraction which is separation dependent. Range-separated functionals such as the ωB97X-D functional^27,28^ include distance-dependent electron–hole attraction within the parametrization of the functional. Here we compare predictions of *G*_*o*_*W*_*o*_@HF/BSE and TDDFT/M06-2X and ωB97X-D methods for both LE and CT states in the DPTZ-DBTO2 emitter, including the effects of excited stated geometry.

Two important effects which are commonly neglected in TADF simulations are the relaxation of the excited state geometry and interactions with the local environment,^4,23,32^ which may be a solvent^33^ or an amorphous or crystalline host. TADF DA and DAD systems exhibit low lying CT excited states where the excited state singlet–triplet splitting, *ΔE*_*ST*_, is small (of order *kT* or less) which facilitates reverse intersystem crossing (rISC)^34^ and an increase in electroluminescence quantum yield.

Excitation energies for optical transitions with a strong CT character are known to be grossly underestimated by TDDFT with local or generalized gradient exchange-correlation functionals.^35,36^ The case of valence versus CT excitations has been studied by TDDFT and *GW*/BSE methods^14,16^ compared to CASPT2 calculations. The cause of the failure of local TDDFT for CT states is the omission of long-range, screened, electron–hole attraction by the local functional.^35^ This can be added to the TDDFT method by replacing the local exchange functional by an exact exchange hybrid functional with a constant weight factor or by a range-separated functional such as CAM-B3LYP^37^ or ωB97X-D in which exact exchange is strongly attenuated at short-range and reaches a fixed fraction of the exact exchange at long-range. In hybrid functionals such as B3LYP (20% exact exchange)^38,39^ or M06-2X (54% exact exchange)^26^ the weight of exact exchange in SCF calculations and electron–hole attraction in TDDFT calculations is independent of electron–hole separation.

In the *GW* approximation, reduction of the proportion of exact exchange is determined by a screened, distance and state-dependent Coulomb interaction, *W*. The *G*_*o*_*W*_*o*_@HF approximation yields a relatively small correction to SCF IP and EA levels, which places them in good agreement with gas phase IP and EA values of organic molecules. Bruneval and co-workers^40^ showed that *G*_*o*_*W*_*o*_@HF predicted slightly higher HOMO–LUMO (H-L) gaps than CCSD(T) for the Thiel set,^41,42^ while *G*_*o*_*W*_*o*_@BHLYPpredicted slightly lower gaps than CCSD(T). Density functionals with lower exact exchange weights than BHLYP (50% exact exchange)^39,43^ predicted lower H-L gaps and poorer agreement with CCSD(T), suggesting that the *G*_*o*_*W*_*o*_@HF starting point adopted in this work is a reasonable starting point for gas phase *GW* calculations for singlet excited states. However, it is known that mean absolute and mean signed errors in *GW*/BSE predictions of triplet excitation energies are larger than those for singlets.^13,40^ Electrochemical measurements show that gas phase IP and EA values are significantly modified in solution owing to screening by solvent molecules, and we use a conductor-like polarizable continuum model (C-PCM)^44^ to estimate shifts in IP and EA values in solution.

Linear response methods for excited states including TDDFT, BSE, and configuration interaction singles (CIS) are readily applied to both singlet and triplet excited states. Singlet state Hamiltonians contain both electron–hole attraction and electron–hole scattering terms, while triplet states contain only the former. In a single-particle excitation picture, the electron–hole scattering term is the exchange integral which determines singlet–triplet splitting. Inclusion of exact exchange in an SCF calculation increases the H-L gap in molecules and results in more localized orbitals. In donor–acceptor systems, this results in smaller electron–hole scattering (exchange) integrals and smaller singlet–triplet splittings. This is crucial to the TADF systems of interest here as the singlet–triplet splitting energy must be within *kT* in order for rISC to proceed. Predicted singlet and triplet energies in solution or condensed phase environments therefore depend on competing factors that determine the degree of inter and intramolecular screening. This has led to a number of studies that have benchmarked various density functionals^3,29−33,45−48^ and the *GW*/BSE approximation in their abilities to predict singlet^29,32,33^ and triplet^3,45^ excitation energies and singlet–triplet splittings^45,46^ of mainly small organic molecules, mainly against superior but more computationally expensive quantum chemistry methods.

In the following section, we outline the *GW*/BSE method used in this work, we review existing benchmark studies for singlet and triplet excitation energies of organic molecules using *GW*/BSE and TDDFT methods, and we describe the geometry optimization protocols and the basis sets used in this work. Two molecular geometries are used throughout, denoted GS and CT. GS is the ground state geometry of the DPTZ-DBTO2 molecule, CT is the equilibrium geometry of the lowest energy singlet CT excited state. Single-particle energy levels predicted by *G*_*o*_*W*_*o*_@HF/BSE and TDDFT/ωB97X-D and M06-2X methods for the GS and CT geometries are compared, followed by excited state energies and optical absorption and fluorescence spectra at each of the three geometries. This is followed by a discussion of results and a summary.

## II Theory and Computation

### II.A *GW* Approximation

Perturbative *G*_*o*_*W*_*o*_ calculations are commonly performed using DFT Kohn–Sham states and energy eigenvalues as a starting point for calculation of *G*_0_ and *W*_0_. Here, HF states and energy eigenvalues are used. The bare Fock exchange is included in the starting Hamiltonian in this case and does not replace a DFT exchange-correlation potential in the self-energy, as when starting from Kohn–Sham DFT. Rostgaard and co-workers^9^ found that a *G*_*o*_*W*_*o*_@HF approach applied to 34 small molecules yielded the lowest mean absolute error (MAE), 0.4 eV, in the IP, compared to experiment, smaller than the MAE for self-consistent *GW*(0.5 eV) and the PBE0 functional^49−51^ (25% exact exchange, 2.55 eV). Working on 13 larger organic molecules, Blase and co-workers^10^ found that while a DFT-LDA starting point yielded relatively large MAE in IP values for those molecules, (0.47 eV, 6%), employing “HF-like” eigenvalues in the dielectric function yielded improved agreement with experimental IP values (MAE 0.31 eV, 4.0%), similar to self-consistent *GW* values (MAE 0.30 eV, 3.8%) and improved prediction of the H-L gap for three acenes and C_60_ (MAE 0.10 eV, 2%). Application of the Exciton code^52−54^ used in this work to acenes from naphthalene to pentacene yielded MAE of 0.03 eV for the IP values and 0.17 eV for both the EA and H-L gaps (unpublished results). Recent work^13,17^ has shown that PBE0 starting points for *GW* calculations yielded poor singlet excitation energies in a *G*_*o*_*W*_*o*_@PBE0 approximation and that this is improved significantly by partial self-consistency in the *GW*self-energy.

The self-energy operator, Σ(**r**,**r**^′^; ϵ), in many-body theory replaces the exchange correlation potential in DFT or the Fock exchange in HF theory by a dynamically screened exchange interaction. The *G*_*o*_*W*_*o*_ approximation to the self-energy has been widely applied to periodic systems where it is conventional to express the screened interaction, *W*_*o*_, in terms of the random phase approximation (RPA) inverse dielectric function, and bare Coulomb potential.^7^ A formally equivalent approach is to express *W*_*o*_ as,
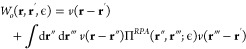
1where *v* is the bare Coulomb potential and Π^*RPA*^ is the RPA polarizability.^55^

Linear response many-body approximations such as RPA, TDHF, BSE or TDDFT can be expressed as the following generalized eigenvalue problem,
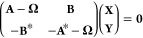
2where the matrices **A** and **B** contain HF, DFT, or *G*_*o*_*W*_*o*_ energy eigenvalues and the electron repulsion integrals shown in Table 1. Its eigenvectors contain excitation, **X**, and deexcitation amplitudes, **Y**, and diagonal eigenvalue matrices, **Ω**, contain pairs of eigenvalues with opposite sign, Ω_±_. The RPA polarizability matrix is constructed from sums of excitation and deexcitation amplitudes and eigenvalues,
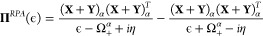
3ϵ, α, and η are the energy of an external field, a label for excited states, and a positive infinitesimal, respectively. Omission of the coupling **B** matrices in eq 2 is known as the Tamm–Dancoff approximation (TDA).^56,57^ In this work, RPA calculations used to calculate the screened interaction, *W*_*o*_, are performed without the TDA, and BSE and TDDFT calculations are performed with the TDA.

**Table 1:** Table of Matrix Elements of *A* and *B* Blocks of the Hamiltonian for RPA, BSE, TDDFT, and TDHF Approximations for a Spin Singlet State^a^

Method	
RPA A	(ϵ_*a*_ – ϵ_*i*_)δ_*ij*_δ_*ab*_ + 2(*ai*|*jb*)
RPA B	2(ai|bj)
BSE A	(ϵ_*a*_~ – ϵ_*i*_~) + 2(*ai*|*bj*) – (*ab*|*W*_*d*_|*ji*)
BSE B	2(*ai*|*bj*) – (*aj*|*W*_*d*_|*bi*)
TDHF A	(ϵ_*a*_ – ϵ_*i*_)δ_*ij*_δ_*ab*_ + 2(*ai*|*jb*) – (*ab*|*ji*)
TDHF B	2(*ai*|*bj*) – (*aj*|*bi*)
TDDFT A	(ϵ_*a*_~ – ϵ_*i*_~) + 2(*ai*|*bj*) – (*ab*|*f*_*xc*_|*ji*)
TDDFT B	2(*ai*|*bj*) – (*aj*|*f*_*xc*_|*bi*)

a For a spin triplet state the electron-hole scattering terms, which have a factor of 2, are omitted. Factors of 2 arise from summation over spin, tildes on energy eigenvalues for BSE indicate *G*_0_W_0_@HF eigenvalues, and *W*_*d*_ is the dynamic part of the screened interaction, *W*_*o*_. *f*_*xc*_ is the TDDFT exchange-correlation kernel.

The bare Coulomb term on the right-hand side of eq 1 contributes the static HF exchange part of the self-energy and the second term is responsible for dynamic screening. Since we use a noninteracting Green’s function expressed in terms of HF wave functions and orbital energies, this term is already accounted for, and only the second, dynamic screening term needs to be calculated.

Insertion of factors ψ_*a*_(**r**) ψ_i_*(**r**) into the density–density response matrix (eq 3) to yield the RPA polarizability and integration over **r**″ and **r**‴ in eq 1 yields the matrix representation of the screened interaction,
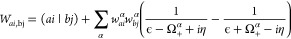
4where
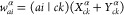
5

### II.B *G*_*o*_*W*_*o*_ Self Energy

Diagonal elements of the self-energy are obtained from convolution of the HF Green’s function with the screened interaction along the real energy axis,
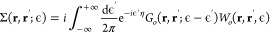
6Residues of the poles of *G*_*o*_ times the bare Coulomb potential in *W*_*o*_ are ψ_*i*_(**r**)ψ_i_*(**r**^′^)*v*(**r** – **r**^′^), and diagonal matrix elements of this term in the self-energy are the HF exchange matrix elements. As noted in the previous section, these are included in the HF Hamiltonian and hence are omitted from the self-energy. Only the latter, dynamic part of the screened interaction contributes to the self-energy. The form of the self-energy in eq 6 is closely analogous to the self-energy obtained using a plasmon-pole approximation to the inverse dielectric function.^7^ However, in the present case, no plasmon pole approximation is required. Residues of *W*_*d*_ contribute the following diagonal matrix elements to the self-energy operator for occupied or virtual states,
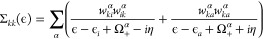
7Finally, the self-energy which is used to calculate shifts in HF eigenvalues is multiplied by the quasi-particle weight function, .

### II.C Solvent Interaction and the H-L Gap

While *G*_*o*_*W*_*o*_@HF yields IP and EA values in reasonable agreement with gas phase experimental values, the H-L gap derived from these values is significantly larger than values estimated from cyclic voltammetry measurements.^34^ We apply a very simple approximation to estimate changes in H and L level positions due to additional solvent screening based on the reduction in IP and increase in EA in the presence of a uniform dielectric with dielectric constant, ε, and cavity radius, *R*, which is given by,
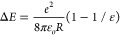
8Calculations of *ΔE* are performed using the conductor-like polarizable continuum model (C-PCM)^44^ in GAMESS.^58,59^

### II.D Bethe–Salpeter Equation and TDDFT Methods

In this work, both BSE and TDDFT calculations are performed within the TDA by solving the ordinary eigenvalue problems which result when the coupling, **B**, matrices in eq 2 are omitted. The distinction between BSE-TDA and TDHF-TDA (or CIS) is that screened electron–hole attraction matrix elements in the BSE **A** matrix in Table 1, (*ab*|*W*_*d*_|*ji*), are replaced by unscreened matrix elements, (*ab*|*ji*), and the *G*_*o*_*W*_*o*_@HF eigenvalue differences, (ϵ̃_*a*_ – ϵ̃_*i*_), by HF eigenvalue differences, (ϵ_*a*_ – ϵ_*i*_). CIS generally overestimates singlet excitation energies,^46^ especially in CT states.^60^ BSE-TDA diagonal element *G*_*o*_*W*_*o*_@HF eigenvalue differences are smaller than HF eigenvalue differences, which reduces excitation energies, while screening of electron–hole attraction matrix elements raises excitation energies. BSE-TDA calculations were performed using the *G*_*o*_*W*_*o*_@HF/BSE module in the Exciton code,^52,53^ TDDFT-TDA calculations were performed using the Gaussian16 code.^61^

The Exciton code uses a density fitting method to calculate four-center integrals. The implementation of density fitting is described in refs (53 and 54). *G*_*o*_*W*_*o*_@HF/BSE calculations for the D and A molecules used all valence and all virtual orbitals in the active space, and for the DAD molecule, they used 70 valence orbitals and 412 virtual orbitals. Increasing the virtual orbital space by an additional 200 orbitals reduced the first excited state energy in the CT geometry by 0.04 eV, indicating that the excitation energies are reasonably well converged with respect to the active space dimension.

### II.E Basis Sets, Geometry Optimization, and Density Functionals

Ground (GS) and (CT) excited state equilibrium geometries were calculated using HF, B3LYP,^38,39^ TPSSh,^62,63^ M06-2X,^26^ and ωB97X-D^27,28^ functionals with cc-pVDZ^64^ or KTZVP^65^ basis sets. Excitation energies at these geometries were calculated using def2-TZVP^66^ or cc-pVDZ basis sets. The effect of dispersion forces on molecular geometries was investigated by adding the D3-BJ functional^67^ to the B3LYP and TPSSh functionals. Bond lengths to heteroatoms in DPTZ-DBTO2 predicted using HF, B3LYP, TPSSh, CIS, TDDFT/B3LYP, and TDDFT/ωB97X-D are given in the Supporting Information, Table S1. HF and M06-2X frontier orbital isosurfaces are shown in the Supporting Information, Figures S1 and S2. Molecular coordinates for the GS and CT geometries used are given in the Supporting Information, Tables S2 to S11.

Singlet and triplet excitation energies for the ground state equilibrium geometries with HF, B3LYP, B3LYP+D3-BJ, TPSSh+D3-BJ, ωB97X-D, and M06-2X functionals using M06-2X and ωB97X-D functionals in the TDDFT calculation are compared in the Supporting Information, Tables S12 and S13. Each of these geometries yielded similar singlet and triplet excitation energies. Excitation energies for the HF ground state and a *GW*/BSE method are also given in Tables S12 and S13.

*G*_*o*_*W*_*o*_@HF/BSE singlet and triplet excitation energies were calculated using the TPSSh + D3-BJ GS geometry obtained using cc-pVDZ basis sets and def2-TZVP basis sets for the BSE calculation and corresponding def2-TZVP-RI auxiliary basis sets^68^ for density fitting Coulomb matrix elements in RPA and BSE *A* matrices. Basis sets were obtained from the EMSL database.^69,70^

The effects of basis set quality, addition of dispersion forces, and choice of DFT exchange-correlation potential on singlet and triplet excitation energies in the GS geometry are presented in the Supporting Information, Tables S12 and S13. The first five excitation energies of each irreducible representation are given for a range of basis sets, inclusion or omission of dispersion forces via the D3-BJ functional and additional functionals used for GS geometry relaxation (HF, TPSSh and B3LYP) are included. Switching from a B3LYP+D3-BJ to a TPSSh+D3-BJ functional for GS geometry relaxation increased the average excitation energy by 0.003 eV for both ωB97X-D and M06-2X TDDFT kernels over all irreducible representations (columns 5 and 6 and columns 10 and 11 in Table S12). Switching from a cc-pVDZ basis to a def2-TZVP basis for a ωB97X-D TDDFT kernel increased the average excitation energy by 0.061 eV (columns 8 and 9). Adding a D3-BJ functional to a B3LYP functional for geometry relaxation in a TDDFT/M06-2X calculation reduced the average excitation energy by −0.024 eV (columns 9 and 10). Using the B3LYP equilibrium geometry versus the HF equilibrium geometry increased the average excitation energy by 0.014 eV. Differences in A_2_ and B_1_ excitation energies on adding the D3-BJ functional to the B3LYP functional in geometry optimization are up to 0.08 eV (Supporting Information, Table S12, columns 9 and 10) with a MAD of 0.028 eV. Adding the D3-BJ functional to the B3LYP functional in geometry optimization reduced the *G*_*o*_*W*_*o*_@HF/BSE 1^1^A_2_ excitation energy by 0.14 eV (Supporting Information, Table S12, columns 1 and 3). The main determinant of excitation energies, however, is the choice of functional in the TDDFT or BSE calculation.

Each of the methods used predicts the lowest singlet excited state of LE character to be a ^1^DD state. A local minimum in the potential energy surface can be found for this state in CIS geometry optimization, but not using any of the TDDFT methods. In that case the optimizing state evolves into a ^1^DA state. The lowest energy ^1^AA acceptor LE state is higher in energy. The CT state is predominantly of H/L character and the ^1^DD LE state is of H/L+2 character. H, L and L+2 orbitals are of a_1_, b_1_, and a_2_ symmetry in the *C*_2*v*_ GS geometry, and therefore, the ^1^DA and ^1^DD states are of B_1_ and A_2_ symmetries. There is a nearly degenerate ^1^DD state of B_1_ symmetry in the GS geometry with H-1/L+2 character, since the H and H-1 levels are nearly degenerate. The CT state in the GS geometry has a nearly degenerate ^1^DA state of A_2_ symmetry. Degeneracies of these states are lifted when the geometry of excited states are optimized and the molecule breaks symmetry. In the CT ^1^DA state equilibrium geometry, the excited hole and electron localize on D and A, respectively, and have large excited state dipole moments.

The choice of hybrid density functional for this work was based on benchmark studies of singlet and triplet states of organic molecule databases against best theoretical estimate values from superior quantum chemistry methods. Jacquemin and co-workers^29^ applied 34 functionals to calculation of triplet excited state energies using def2-TZVP basis sets and found the best functionals to be BMK^71^ and M06-2X with mean signed error (MSE) of 0.18 and 0.07 and mean absolute error (MAE) of 0.24 and 0.23 eV, respectively, compared to best theoretical estimates. In comparison, CIS and M06-HF give −0.06 and 0.12 and 0.44 and 0.56 eV for MSE. Brückner and Engels investigated the effect of a fraction of the Fock exchange on the singlet–triplet splitting^46^ in organic DAD systems using a multistate CASPT2/ccpVTZ method as reference. They concluded that, “the M06-2X functional provides best estimates for both vertical and adiabatic singlet–triplet gaps when adiabatic linear-response TD-DFT singlet excitations are combined with TDA triplet values”. More recent assessments of hybrid functionals have been performed for singlet and triplet excitations^30,48^ from the QUEST database.^72^ Grotjahn and Kaupp^48^ found that the overall best performing global hybrid was M06-2X, with a mean absolute error of 0.20 eV for π to π* triplet vertical excitation energies. Performances of M06-2X and a range of other functionals over a range of excitation types (valence, Rydberg, and charge transfer) were compared by Jacquemin and co-workers.^73^ In many cases M06-2X performs comparatively well, although in some cases, such as transitions in Ni(II) complexes,^74^ it can fail spectacularly.

Liang and co-workers^30^ found that the ωB97X-D^27,28^ and BMK^71^ functionals gave the best overall performance for 463 vertical excitation energies, with a root-mean-square error (RMSE) of 0.27 eV. The RMSE for M06-2X in that study was less than 0.3 eV. Loos and co-workers^31^ reported reference energies for 17 molecules with intramolecular CT states. They found the best performing functional in TDDFT to be ωB97X-D. Bruneval and co-workers^40^ applied a *GW*/BSE approach to singlet and triplet excitation energies of Thiel’s set of organic molecules^41,42^ using a range of DFT, hybrid DFT, and HF starting points. They found a strong, linear correlation between the MSE of the S_1_and T_1_excitation energies predicted by BSE and the H-L gap. The H-L gap is determined by the chosen starting point. No BSE@HF result was reported, but the smallest S_1_ MSE (compared to a best theoretical estimate) was found for BSE@BHLYP, where the starting hybrid functional has an exact exchange weight of 50%. T_1_ excitation energies showed a similar correlation with H-L gap, but the optimal H-L gaps for minimal MSE, in either case, differed by 0.6 eV.

Jacquemin and co-workers^45^ reported singlet and triplet excitation energies and singlet–triplet splittings in 20 organic molecules from TDDFT and *GW*/BSE calculations in which single-particle eigenvalues appearing in *G* and *W* (eq 4) were iterated to self-consistency prior to the BSE calculation. Coupled-cluster (CC3) calculations were used as a benchmark.^41^ The M06 family of hybrid functionals^26^ were used as starting points. TDDFT-TDA with the M06-2X functional gave a MSE of 0.00 eV and a MAE of 0.20 for triplet excitation energies, compared to CC3 using a TZVP basis (also used for *G*_*o*_*W*_*o*_@HF/BSE here). By comparison, the M06-HF functional (100% exact exchange) gave a MSE of 0.20 eV and a MAE of 0.44 eV in TDDFT-TDA. BSE-TDA using the M06-2X functional gave a MSE of −0.31 eV and a MAE of 0.31 eV. The more localized wave functions in a M06-HF starting point gave corresponding errors of −0.06 and 0.17 eV, using the self-consistent eigenvalue *GW* approach.

Turning to singlet–triplet splitting energies calculated using TZVP basis sets and compared to CC3, Jacquemin and co-workers^45^ found similar MSE and MAE values for both M06-2X and M06-HF starting points. The MSE and MAE using a M06-2X starting point and TDDFT-TDA were 0.42 and 0.43 eV, respectively, and for BSE-TDA they were 0.31 and 0.31 eV. These results give some context for the triplet energies and singlet–triplet splittings reported in Section III.B. TDDFT/M06-2X and *GW*/BSE with a M06-HF starting point iterated to self-consistency in the *GW* calculation had rather similar MSE and MAE for triplet excitations (0.00 versus −0.06 eV and 0.20 versus 0.17 eV). Errors in singlet–triplet splittings were smaller by 0.1 eV in BSE-TDA.

## III Results

### III.A Single Particle Energy Levels

In this section, we compare single particle energies from *G*_*o*_*W*_*o*_@HF, ωB97X-D, and M06-2X to ΔSCF calculations for those methods. We also consider the effect of polar and nonpolar solvent polarization on single particle levels using the C-PCM model and compare to H and L energies from cyclic voltammetry.

**Figure 2:**
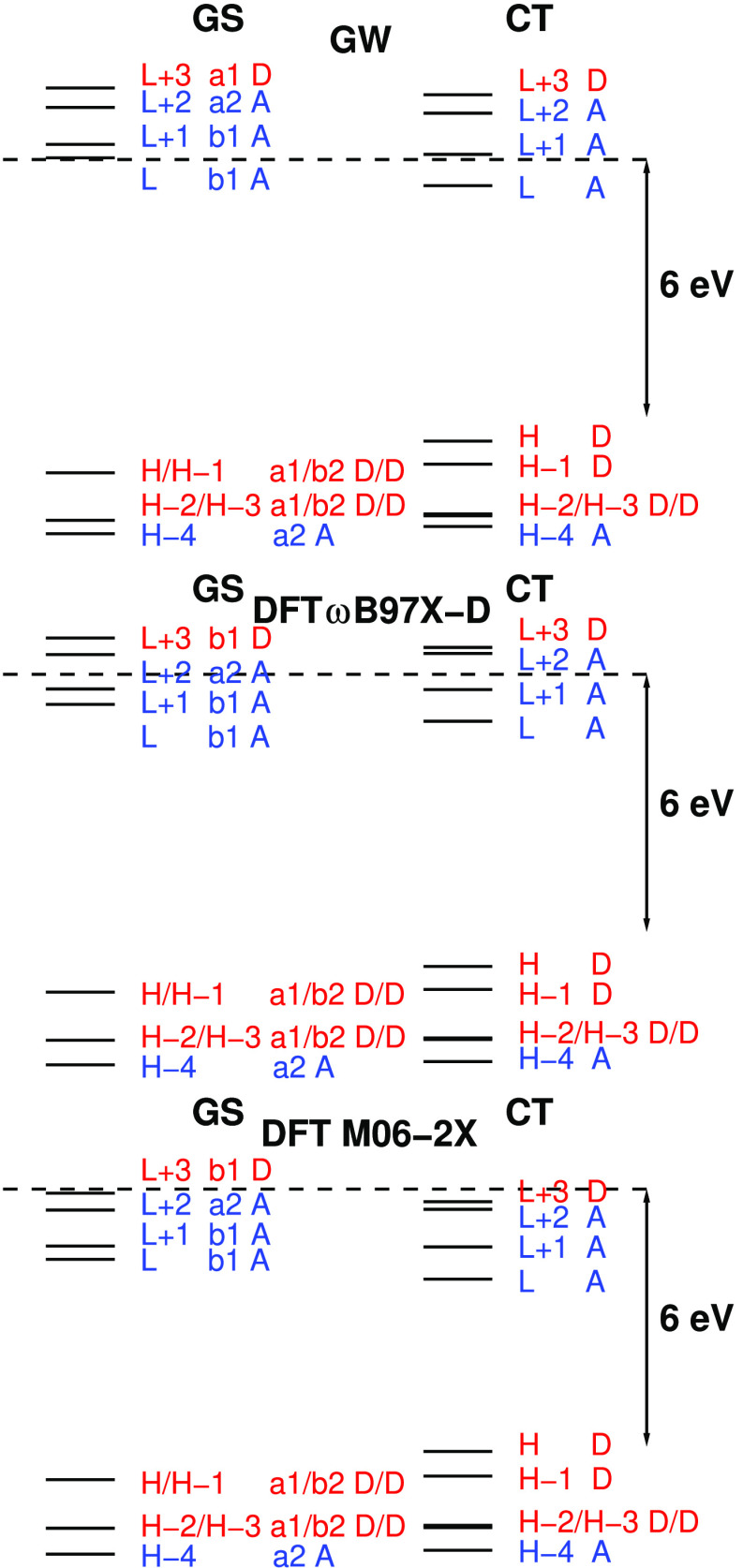
Single particle levels for DPTZ-DBTO2 from *G*_*o*_*W*_*o*_@HF and DFT/ωB97X-D and DFT/M06-2X calculations using the GS and CT geometries. *C*_2*v*_ MO symmetries are given for the GS geometry and MO localization on donor and acceptor is indicated by D or A. The vacuum level is indicated by a dotted line.

Single particle energy levels from *G*_*o*_*W*_*o*_@HF, ωB97X-D, and M06-2X calculations for DPTZ-DBTO2 in the GS and CT geometries are shown in Figure 2. In each case the highest four occupied orbitals are localized on D fragments and the fifth on the A fragment (see Supporting Information, Figures S1 and S2, for isosurface plots of HF and M06-2X Kohn–Sham orbitals). The first three virtual orbitals are localized on A and the fourth and fifth on D fragments in the GS and CT geometries. Supporting Information Figure S1 shows that the L+4 HF orbital in the GS geometry becomes the L+3 orbital in the CT geometry. The L+2 orbital is mainly localized on the A fragment in the GS and CT geometries and partly on the D fragments.

In the GS geometry, weak interaction between linear combinations of D orbitals produces quasi-degenerate a_1_, b_2_ orbital pairs (H/H-1 and H-2/H-3) localized on the D fragments. Relaxation of the structure in the CT geometry lifts the near degeneracy of the H/H-1 pair. These MOs localize on either D fragment and the quasi-degenerate H and H-1 a_1_, b_2_ pair of levels splits by 0.5 eV in *G*_*o*_*W*_*o*_@HF and ωB97X-D and by 0.6 eV in M06-2X. The L+4 and L+5 levels are also localized on D fragments and are nearly degenerate with L+3. There is reordering of levels in the GS geometry between HF and DFT. In HF, L+4 is of b_1_ symmetry, and in DFT L+5, it is of b_1_ symmetry.

Ionization potentials (IP), electron affinities (EA), and IP–EA differences from SCF HF, *G*_*o*_*W*_*o*_@HF, ωB97X-D, and M06-2X eigenvalues and from ΔSCF calculations are given in Table 2. HF eigenvalue differences overestimate the IP and underestimate the EA obtained from ΔSCF calculations, as expected. HF eigenvalue differences overestimate the ΔSCF IP values for GS and CT geometries by 0.46 and 1.31 eV, respectively. ΔSCF EA values are underestimated by 1.31 and 1.33 eV and the *E*_*G*_ gap is overestimated by 1.77 and 2.63 eV, respectively. These differences are reduced in *G*_*o*_*W*_*o*_@HF. The IP is overestimated by 0.08 and 0.76 eV, the EA is underestimated by 0.33 and 0.25 eV and the *E*_*G*_ gap is overestimated by 0.41 and 1.00 eV for the GS and CT geometries, respectively. The ωB97X-D functional overestimates the IP by 0.10 and 0.26 eV, underestimates the EA by 0.34 and 0.33 eV, and overestimates *E*_*G*_ by 0.45 and 0.59 eV for the GS and CT geometries. In contrast, the M06-2X functional underestimates the IP by 0.46 and 0.51 eV and overestimates the EA by 0.47 and 0.46 eV, leading to underestimates of the *E*_*G*_ gap by 0.93 and 0.98 eV in the GS and CT geometries, respectively.

**Table 2:** Ionization Potential (IP), Electron Affinity (EA), and IP–EA Energy Gaps (*E*_*G*_) in eV from *G*_*o*_*W*_*o*_@HF, ωB97X-D, and M06-2X Single Particle Energy Levels and IP, EA ,and *E*_*G*_ gaps from ΔSCF Calculations for the GS and CT Geometries

	GS/IP	CT/IP	GS/EA	CT/EA	GS/*E*_*G*_	CT/*E*_*G*_
HF	7.68	7.11	–1.02	–0.47	8.70	7.58
*G*_*o*_*W*_*o*_@HF	7.30	6.56	–0.04	0.61	7.34	5.95
ΔSCF HF	7.22	5.80	0.29	0.86	6.93	4.95
ωB97X-D	7.41	6.81	0.70	1.09	6.71	5.72
ΔSCF ωB97X-D	7.31	6.41	1.04	1.19	6.26	5.22
M06-2X	6.77	6.12	1.64	2.10	5.13	4.02
ΔSCF M06-2X	7.23	6.63	1.17	1.64	6.06	5.00

One of the challenges for prediction of relative excitation energies of CT versus LE states is the physical difference in exciton binding in the CT state versus the LE state, owing to the localization of electron and hole on the same or different molecular fragments. An unscreened Coulombic interaction energy is approximately 14.4/*r* eVÅ^–1^, where *r* is the electron–hole separation. Since the electron–hole separation is significantly larger in a CT transition and the Coulombic interaction energy is comparable to excitation energies, interaction screening in *G*_*o*_*W*_*o*_@HF and ωB97X-D or truncation in M06-2X DFT calculations determines the relative cost of CT and LE excitations. Table 3 shows DD, AA, and DA gaps and DD - DA gap differences for each method for both geometries. There is agreement in DD–DA gap differences in each geometry. The “energetic advantage” of DA over DD noninteracting excitations ranges from 1.54 eV in the GS geometry with M06-2X to 1.99 eV in the CT geometry using *G*_*o*_*W*_*o*_@HF. When electron and hole separate to a larger degree in the CT state, this advantage overcomes the energetic cost of separating the electron and hole to a larger extent in the CT state and results in lowest energy CT and LE excitations of similar energies.

**Table 3:** Single Particle Energy Gaps in eV between States of D and A Character around the HOMO–LUMO Gap from *G*_*o*_*W*_*o*_@HF, ωB97X-D, and M06-2X for the GS and CT Geometries

	GS/DD	GS/AA	GS/DA	GS/DD-DA
*G*_*o*_*W*_*o*_@HF	8.97	8.76	7.34	1.63
ωB97X-D	8.26	8.40	6.71	1.55
M06-2X	6.67	6.87	5.13	1.54

	CT/DD	CT/AA	CT/DA	CT/DD-DA
*G*_*o*_*W*_*o*_@HF	8.07	7.94	5.95	1.99
ωB97X-D	7.44	7.93	5.72	1.72
M06-2X	5.82	6.32	4.02	1.80

Finally, in this section, we consider the effect of dielectric screening by the solvent on H and L levels of DPTZ-DBTO2. Dias et al. reported electrochemical measurements of the redox potentials of the PTZ donor, DBTO2 acceptor, and DPTZ-DBTO2 DAD system,^34^ from which they estimated the H levels in the D and DAD systems to be −5.2 and −5.4 eV and the L levels in the A and DAD systems to be −2.9 and −3.1 eV, respectively. Measurements were performed in a 0.1 M solution of Bu_4_NBF_4_ in dichloromethane(DCM). We present results of conductor-like PCM calculations using DCM and cyclohexane (CHX) as polar and nonpolar solvents. Polar and nonpolar solvents differ in that only the former have a large screening contribution from permanent dipole reorientation. These calculations are intended to give an indication of the magnitude of solvent induced shifts.

Redox potentials are successfully reproduced in first-principles calculations which use protocols where the free energies of geometry-relaxed solvated complexes are computed.^75,76^ In an equilibrium electrochemical ionization process, where dipole reorientation and electronic polarization screen the ion formed lowering of the IP and raising of the EA will be larger than in a fast process, such as optical excitation, where dipole reorientation is generally too slow to contribute to screening. Changes in IP and EA values estimated from C-PCM calculations using DCM and CHX therefore represent limits where electronic plus dipole orientation and electronic polarization only are included.

Differences in electrostatic interaction energies for solvated ionic and neutral species from conductor-like PCM calculations using the M06-2X functional in GAMESS^44^ for the D, A, and DAD systems in cyclohexane (CHX, ε = 2.02) and DCM (ε = 8.93) are given in Table 4. Mean radii derived from cavity volumes reported by GAMESS for calculations for D, A and DAD systems are 3.56, 3.49, and 4.89 Å, respectively. When these radii are used in eq 8 together with these ε values the C-PCM changes in electrostatic solvation energy are reproduced fairly well. C-PCM values in Table 4 are used to estimate the H and L energy level shifts in a dielectric solvent relative to gas phase IP and EA values in Table 5. Internal and Gibbs free energies of ion solvation in a polar solvent exceed 1 eV.^77^ M06-2X H and L levels for the DAD system in the gas phase and the CT geometry are −6.12 and −2.10 eV compared to estimates of these levels from cyclic voltammetry of −5.4 and −3.1 eV.^34^ Using the simple estimates given in Table 4, the M06-2X DAD H level shifts up by 1.49 eV in DCM leaving it 0.8 eV above the experimental H level estimate; the DAD L level shifts down by 1.29 eV in DCM, leaving it 0.3 eV below the experimental value and giving an H-L gap of 1.24 eV. DCM is relatively polar. A large proportion of the estimated shift due to electrostatic interaction is via slow molecular dipole reorientation. CHX is nonpolar and is therefore typical of high frequency electronic polarization only. Smaller estimated shifts from CHX, due to electronic polarization alone, yield estimates for the H level which is within 0.1 eV of the experimental estimate, and the L level is 0.2 eV above the experimental estimate and the estimated gap is 2.40 eV.

**Table 4:** Changes in Electrostatic Solvation Energies in eV on Going from Neutral to Ionic Species^a^

solvent	D H	A L	DAD H	DAD L
CHX	0.82 (1.02)	0.88 (1.04)	0.81 (0.74)	0.69 (0.74)
DCM	1.44 (1.81)	1.59 (1.84)	1.49 (1.32)	1.29 (1.32)

a Values are predicted by conductor-like PCM calculations on PTZ (D), DBTO2 (A), and DPTZ-DBTO2 (DAD) in CHX and DCM solvents. Simple estimates using eq 8 and mean cavity radii from the C-PCM calculations are given in brackets. Positive or negative charge states are indicated by H or L when ionization is of the HOMO or LUMO level.

**Table 5:** DPTZ-DBTO2 (DAD) H and L Energies and H-L Energy Gaps for the CT Geometry in eV from *G*_*o*_*W*_*o*_@HF, DFT/ωB97X-D, and DFT/M06-2X Calculations in the Gas Phase (GAS) or with CHX and DCM Solvents in a C-PCM Model and H and L Energies from Cyclic Voltammetry Measurements Performed in DCM^34^ a

method	DAD H	DAD L	DAD H–L
expt(DCM)	–5.4	–3.1	2.3
*G*_*o*_*W*_*o*_@HF(GAS)	–6.56	–0.61	5.95
*G*_*o*_*W*_*o*_@HF(CHX)	–5.75	–1.30	4.45
*G*_*o*_*W*_*o*_@HF(DCM)	–5.07	–1.90	3.17
ωB97X-D(GAS)	–6.81	–1.09	5.72
ωB97X-D(CHX)	–6.00	–1.78	4.22
ωB97X-D(DCM)	–5.32	–2.38	2.94
M06-2X(GAS)	–6.12	–2.10	4.02
M06-2X(CHX)	–5.31	–2.91	2.40
M06-2X(DCM)	–4.63	–3.39	1.24

a Experimental uncertainty was estimated to be ±0.1 eV.

Applying the same estimated shifts to *G*_*o*_*W*_*o*_@HF gas phase H and L levels (−6.56 and −0.61 eV in DAD) and ωB97X-D gas phase H and L levels (−6.81 and −1.09 eV) with CHX solvent results in H levels 0.4 and 0.6 eV below the experimental value and L levels 1.8 and 1.3 eV above the experimental estimates from cyclic voltammetry.^34^ With a DCM solvent the IP value is overestimated by *G*_*o*_*W*_*o*_@HF by 0.3 eV and within 0.1 eV (ωB97X-D). The EA value is underestimated by 1.2 eV by *G*_*o*_*W*_*o*_@HF and within 0.1 eV of experiment according to ωB97X-D.

### III.B Excited State Energies

In this section, we consider the character of low-lying singlet and triplet excited states for the GS and CT geometries, their relative stabilities and singlet–triplet splittings. As noted in Section III.A, frontier molecular orbitals in DPTZ-DBTO2 localize predominantly on either the D or A fragments of the molecule. Excited states which are dominated by a single excitation can therefore be divided into donor–donor (DD), acceptor–acceptor (AA), and donor–acceptor (DA) transitions. This is the case for some low energy excitations in DPTZ-DBTO2. Local exciton and charge transfer excited states are distinguished by the degree of charge transfer in the excited state. While charge transfer excited states have a small transition dipole moment, they have large excited state permanent dipole moments. DD and AA states typically have excited state dipole moments up to 5 D (of similar magnitude to the GS geometry ground state moment of 4.51 D). In contrast, DA excited states have permanent dipole moments which may exceed 10 D. Comparison of excited state wave functions from the three methods used in this work shows that trends in properties such as excited state wave function coefficients, excitation energies, etc. exist with *G*_*o*_*W*_*o*_@HF/BSE at one end and TDDFT/M06-2X at the other. This may reflect the strength of electron–hole interaction in these methods. This is likely to be greatest in *G*_*o*_*W*_*o*_@HF/BSE, followed by TDDFT/ωB97X-D and TDDFT/M06-2X. However, the different ways in which the Coulombic electron–hole attraction is attenuated by screening approximations in these methods should be kept in mind when comparing electron–hole interaction strengths in LE and CT states.

**Figure 3:**
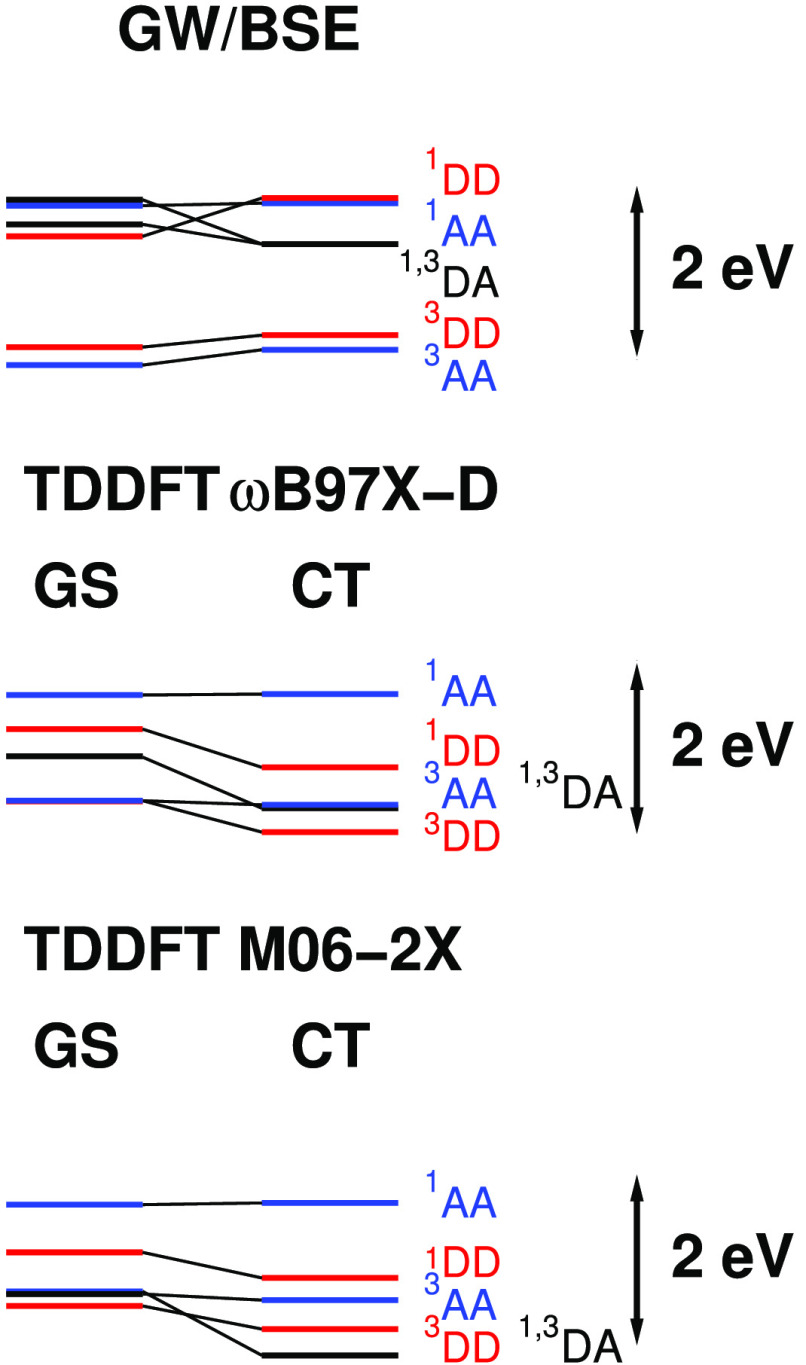
Lowest singlet and triplet excited state energies with DD, AA, and DA character for DPTZ-DBTO2 in GS and CT geometries relative to HF or DFT GS energies. DD, AA, and DA levels are shown in red, blue, and black, respectively.

The lowest 10 singlet and 10 triplet transition energies for the GS and CT geometries predicted by *G*_*o*_*W*_*o*_@HF/BSE, TDDFT/ωB97X-D, and TDDFT/M06-2X are given in Tables 6–9. Where the character of the excited state is clear from the dominant configurations in the excited state and magnitude of the excited state dipole moment, it is marked next to the excitation energy in these tables. The most stable excited states in each geometry and method which are marked in bold in these tables are illustrated schematically in Figure 3. Note that the difference in CT and GS SCF energies (0.59 eV for *G*_*o*_*W*_*o*_@HF, 0.35 eV for ωB97X-D, and 0.40 eV for M06-2X) has been added to the transition energies in Tables 6–9 in drawing Figure 3, so that the levels represent the relative stabilities of these excited states. DA excited state energies, dipole moments, and singlet–triplet splittings are given in Table 10.

**Table 6:** Energies in eV, Excited State Dipole Moments in Debye, and Excited State Transition Type (TT = DD, DA, or AA) of the Ten Lowest Singlet States of DPTZ-DBTO2 in the GS Geometry from *G*_*o*_*W*_*o*_@HF/BSE-TDA(BSE), ωB97X-D(B97), and M06-2X TDDFT-TDA (M06)^a^

GS/BSE			GS/B97			GS/M06		
*E* (eV)	μ (D)	TT	*E* (eV)	μ (D)	TT	*E* (eV)	μ (D)	TT
**4.01**	**5.01**	**DD**	**3.90**	**7.92**	**DA**	**3.60**	**9.12**	**DA**
**4.01**	**4.89**	**DD**	**3.91**	**7.64**	**DA**	**3.61**	**9.16**	**DA**
**4.38**	**6.43**	**AA**	3.91	4.78		3.74	6.52	DA
**4.45**	**11.85**	**DA**	3.95	4.99		3.75	6.48	DA
**4.46**	**9.88**	**DA**	**4.23**	**3.94**	**DD**	**4.09**	**2.26**	**DD**
4.51	3.95		**4.23**	**4.04**	**DD**	**4.09**	**2.32**	**DD**
4.52	3.96		4.60	1.16		4.58	1.09	
4.52	14.83	DA	4.60	1.15		4.58	1.09	
4.56	16.21	DA	**4.63**	**2.58**	**AA**	**4.65**	**2.51**	**AA**
4.59	3.52		4.76	2.06		4.72	2.25	

a States shown in Figure 3 are in bold.

**Table 7:** Energies in eV, Excited State Dipole Moments in Debye, and Excited State Transition Type (TT = DD, DA, or AA) of the Ten Lowest Triplet States of DPTZ-DBTO2 in the GS Geometry from *G*_*o*_*W*_*o*_@HF/BSE-TDA(BSE), ωB97X-D(B97), and M06-2X TDDFT-TDA (M06)^a^

GS/BSE			GS/B97			GS/M06		
*E* (eV)	μ (D)	TT	*E* (eV)	μ (D)	TT	*E* (eV)	μ (D)	TT
**2.50**	**4.77**	**AA**	**3.38**	**1.57**	**DD**	**3.46**	**2.33**	**DD**
**2.71**	**4.68**	**DD**	**3.38**	**1.57**	**DD**	**3.46**	**2.27**	**DD**
**2.71**	**4.68**	**DD**	**3.39**	**1.72**	**AA**	**3.59**	**9.01**	**DA**
3.00	4.80		3.80	1.52		**3.60**	9.08	**DA**
3.00	4.80		3.80	1.52		**3.63**	1.69	**AA**
3.16	4.89		**3.88**	7.41	**DA**	3.78	5.45	DA
3.44	3.89		**3.88**	6.95	**DA**	3.79	5.46	DA
3.44	3.89		3.92	4.53		3.89	1.26	
3.50	5.20		3.94	4.94		3.89	1.26	
3.51	4.57		4.02	1.19		4.20	1.56	

a States shown in Figure 3 are in bold.

**Table 8:** Energies in eV, Excited State Dipole Moments in Debye, and Excited State Transition Type (TT = DD, DA, or AA) of the Ten Lowest Singlet States of DPTZ-DBTO2 in the CT Geometry from *G*_*o*_*W*_*o*_@HF/BSE-TDA(BSE), ωB97X-D(B97), and M06-2X TDDFT-TDA (M06)^a^

CT/BSE			CT/B97			CT/M06		
*E* (eV)	μ (D)	TT	*E* (eV)	μ (D)	TT	*E* (eV)	μ (D)	TT
**3.28**	**15.58**	**DA**	**2.95**	**10.65**	**DA**	**2.53**	**12.99**	**DA**
**3.35**	**17.87**	**DA**	**3.43**	**3.89**	**DD**	3.15	12.97	DA
**3.81**	**5.96**	**AA**	3.61	11.75	DA	3.17	9.72	DA
3.82	24.47	DA	3.62	8.70	DA	**3.39**	**4.45**	**DD**
**3.87**	**8.77**	**DD**	3.87	5.98	DD	3.63	9.08	DA
3.88	14.05	DA	4.13	4.43	DD	3.97	2.32	DD
4.06	4.28		4.21	1.57		4.13	1.78	
4.20	3.54		**4.29**	**2.08**	**AA**	4.21	4.91	
4.26	10.93		4.31	2.58		**4.27**	**2.39**	**AA**
4.37	11.30		4.58	1.03		4.31	15.46	

a States shown in Figure 3 are in bold.

**Table 9:** Energies in eV, Excited State Dipole Moments in Debye, and Excited State Transition Type (TT = DD, DA, or AA) of the Ten Lowest Triplet States of DPTZ-DBTO2 in the CT Geometry from *G*_*o*_*W*_*o*_@HF/BSE-TDA(BSE), ωB97X-D(B97), and M06-2X TDDFT-TDA (M06)^a^

CT/BSE			CT/B97			CT/M06		
*E* (eV)	μ (D)	TT	*E* (eV)	μ (D)	TT	*E* (eV)	μ (D)	TT
**2.09**	**4.63**	**AA**	**2.67**	**3.15**	**DD**	**2.48**	**11.26**	**DA**
**2.26**	**4.94**	**DD**	**2.99**	**1.44**	**AA**	**2.79**	**4.06**	**DD**
2.69	4.63		**3.01**	**9.34**	**DA**	**3.13**	**1.66**	**AA**
2.90	4.84		3.31	1.74		3.13	12.18	DA
3.00	4.71		3.40	1.64		3.19	10.55	DA
3.12	4.86		3.48	6.70		3.38	3.18	
3.19	7.23		3.61	11.60	DA	3.39	1.83	
3.19	5.18		3.77	5.94		3.65	7.65	
**3.33**	**23.82**	**DA**	3.78	1.43		3.69	3.49	
3.38	4.66		3.84	5.18		3.84	1.27	

a States shown in Figure 3 are in bold.

**Table 10:** Excited State Energies Relative to the HF or DFT Ground State Energy in eV, Singlet–Triplet Splittings, Δ*E*_ST_, Predicted by *G*_*o*_*W*_*o*_@HF/BSE(BSE), TDDFT/ωB97X-D(B97), and TDDFT/M06-2X (M06) in meV, and Excited State Permanent Dipole Moments, μ, in Debye for GS and CT Geometries of DPTZ-DBTO2

geom	state	*E* (eV)	μ (D)	state	*E* (eV)	μ (D)	Δ*E*_ST_
GS/BSE	1^1^DA	4.4475	11.85	1^3^DA	4.1558	17.58	291.7
GS/BSE	2^1^DA	4.4644	9.88	2^3^DA	4.1653	17.10	299.1
GS/B97	1^1^DA	3.8977	7.92	1^3^DA	3.8798	7.41	17.9
GS/B97	2^1^DA	3.9067	7.64	2^3^DA	3.8880	6.95	17.9
GS/M06	1^1^DA	3.5960	9.12	1^3^DA	3.5910	9.01	5.0
GS/M06	2^1^DA	3.6085	9.16	2^3^DA	3.6030	9.08	5.5
GS/M06	3^1^DA	3.7417	6.52	3^3^DA	3.7820	5.45	–40.3
GS/M06	4^1^DA	3.7487	6.48	4^3^DA	3.7892	5.46	–40.5
CT/BSE	1^1^DA	3.3584	17.87	1^3^DA	3.3330	23.82	25.4
CT/BSE	2^1^DA	3.8201	24.47	2^3^DA	3.8108	25.84	9.3
CT/B97	1^1^DA	2.9462	10.65	1^3^DA	3.0073	9.34	–61.1
CT/B97	2^1^DA	3.6143	11.75	2^3^DA	3.6082	11.60	6.1
CT/M06	1^1^DA	2.5291	12.99	1^3^DA	2.4789	11.26	50.2
CT/M06	2^1^DA	3.1523	12.97	2^3^DA	3.1293	12.18	23.0
CT/M06	3^1^DA	3.1662	9.72	3^3^DA	3.1899	10.55	–23.7
CT/M06	4^1^DA	3.6305	9.08	4^3^DA	3.6537	7.65	–23.2

#### III.B.1 Vertical Excited States

Inspection of the levels illustrated in Figure 3 shows that predictions of the relative ordering of excited state levels by TDDFT/ωB97X-D and TDDFT/M06-2X are largely in agreement. These functionals place the lowest ^1^DA level below the ^1^DD level in the GS geometry. The singlet–triplet splitting for this DA level is too small to be shown in Figure 3. The ^1^AA level lies above these levels. *G*_*o*_*W*_*o*_@HF/BSE places the lowest ^1^DA level just above the lowest ^1^DD level and the ^1^AA level above these levels. Changes in excited state wave function character for these levels can be seen in the top half of Table 11. The ^1^DD excited state wave function differs between the BSE and TDDFT methods, while the wave functions of the ^1^DA and ^1^AA states change steadily from BSE to M06-2X.

**Table 11:** Leading Pair of Wave Function Components of Singlet and Triplet Excited States and Excitation Energies in eV for GS DPTZ-DBTO2 Predicted by *G*_*o*_*W*_*o*_@HF/BSE, TDDFT/ωB97X-D, and TDDFT/M06-2X

	*E* (eV)	1^1^DD	*E* (eV)	1^1^DA	*E* (eV)	1^1^AA
BSE	4.01	H-0/L+4:0.57 H-1/L+2:0.50	4.46	H-0/L+0:0.28 H-0/L+1:0.44	4.38	H-4/L+0:0.70 H-4/L+1:0.48
B97	4.23	H-0/L+3:0.39 H-1/L+4:0.38	3.90	H-0/L+0:0.49 H-0/L+1:0.31	4.63	H-4/L+0:0.48 H-4/L+1:0.36
M06	4.09	H-0/L+3:0.45 H-1/L+4:0.43	3.60	H-0/L+0:0.65 H-0/L+1:0.14	4.65	H-4/L+0:0.43 H-4/L+1:0.42

	*E* (eV)	1^3^DD	*E* (eV)	1^3^DA	*E* (eV)	1^3^AA
BSE	2.71	H-0/L+4:0.52 H-1/L+6:0.41	4.15	H-0/L+0:0.78 H-0/L+1:0.27	2.50	H-4/L+0:0.75 H-9/L+1:0.33
B97	3.38	H-0/L+3:0.42 H-1/L+4:0.37	3.88	H-0/L+0:0.46 H-0/L+1:0.30	3.39	H-4/L+0:0.58 H-8/L+0:0.24
M06	3.46	H-0/L+3:0.38 H-1/L+4:0.36	3.59	H-0/L+0:0.61 H-0/L+1:0.20	3.63	H-4/L+0:0.61 H-0/L+1:0.20

TDDFT/ωB97X-D predicts the lowest triplet state in the GS geometry to be the ^3^AA state at 3.38 eV with two ^3^DD states slightly above it at 3.38 and 3.39 eV and the ^3^DA state at 3.88 eV. TDDFT/M06-2X predicts the ^3^DD state to be the lowest GS excited state at 3.46 eV, with the ^3^AA state at 3.59 eV and the ^3^DA state at 3.78 eV. Gibson and co-workers^22^ reported a similar ordering of excited state levels at the equilibrium geometry of the PTZ-DBTO2 DA system using the same M06-2X TDDFT functional. They found the S_1_, T_1_ and T_2_ energies at 3.55, 3.45, and 3.53 eV. These compare to energies for the GS geometry from Tables 6 and 7 of 3.60, 3.46, and 3.59 eV.

*G*_*o*_*W*_*o*_@HF/BSE predicts the ^3^AA state to be the lowest at 2.50 eV, with two ^3^DD states at 2.71 eV and the ^3^DA state at 4.15 eV. There are small changes in wave function coefficients for singlet or triplet states predicted by TDDFT/ωB97X-D or TDDFT/M06-2X (Tables 11 and 12) for the ^1,3^DD, ^1,3^DA, and ^1,3^AA states and larger changes going from singlet to triplet states in *G*_*o*_*W*_*o*_@HF/BSE. Relative weights of H/L and H/L+1 configurations in the ^1^DA states change abruptly on going from *G*_*o*_*W*_*o*_@HF/BSE to M06-2X, while they remain similar in the corresponding triplet states. This may result from stronger mixing of ^1^DD and ^1^DA states of B_1_ and A_2_ symmetry in *G*_*o*_*W*_*o*_@HF/BSE where they are closely spaced and little mixing in the triplet states, where the ^3^DD and ^3^DA states are energetically well separated.

**Table 12:** Leading Pair of Wave Function Components of Singlet and Triplet Excited States and Excitation Energies in eV for CT DPTZ-DBTO2 Predicted by *G*_*o*_*W*_*o*_@HF/BSE, TDDFT/ωB97X-D, and TDDFT/M06-2X

	*E* (eV)	1^1^DD	*E* (eV)	1^1^DA	*E* (eV)	1^1^AA
BSE	3.35	H-0/L+0:0.66 H-0/L+2:0.48	3.28	H-0/L+0:0.68 H-0/L+3:0.45	4.20	H-4/L+0:0.89 H-4/L+1:0.23
B97	3.43	H-0/L+3:0.53 H-0/L+1:0.30	2.95	H-0/L+0:0.65 H-0/L+3:0.19	4.29	H-4/L+0:0.63 H-4/L+1:0.18
M06	3.39	H-0/L+3:0.63 H-0/L+1:0.24	2.53	H-0/L+0:0.69 H-0/L+3:0.10	4.27	H-4/L+0:0.63 H-4/L+1:0.21

	*E* (eV)	1^3^DD	*E* (eV)	1^3^DA	*E* (eV)	1^3^AA
BSE	2.26	H-0/L+3:0.65 H-0/L+2:0.50	3.33	H-0/L+0:0.85 H-0/L+11:0.21	2.09	H-4/L+0:0.85 H-9/L+1:0.23
B97	2.67	H-0/L+3:0.58 H-0/L+0:0.32	3.01	H-0/L+0:0.56 H-0/L+3:0.25	2.99	H-4/L+0:0.63 H-8/L+0:0.14
M06	2.79	H-0/L+3:0.54 H-0/L+2:0.28	2.48	H-0/L+0:0.65 H-0/L+3:0.24	3.13	H-4/L+0:0.65 H-8/L+0:0.17

The triplet instability problem in TDDFT^78^ has been analyzed for excited states with LE, CT, and Rydberg character.^79^ It is more severe in excited states with LE character for functionals that contain a higher weight of exact exchange.^79^ It is alleviated to some degree by making the TDA and using range-separated functionals such as ωB97X-D.^79^ Both ωB97X-D and M06-2X TDDFT kernels predict higher triplet excitation energies than *G*_*o*_*W*_*o*_@HF/BSE by between 0.2 and 0.9 eV. Mean signed errors for triplet state energies in the QuestDB benchmark set predicted by ωB97X-D and M06-2X TDDFT are −0.165 and −0.095 eV,^30^ reinforcing the conclusion that *G*_*o*_*W*_*o*_@HF/BSE triplet excited state energies are affected by the triplet instability problem.

In order to compare predictions of triplet excitation energies by *G*_*o*_*W*_*o*_@HF/BSE and the two TDDFT methods, averages of the first five triplet excitation energies of each symmetry type in the GS geometry were calculated for each method. Differences in these averages between the *G*_*o*_*W*_*o*_@HF/BSE method and the two TDDFT methods are given in Table 13. For example, the average of the first five singlet excitation energies of A_1_ symmetry predicted by TDDFT/ωB97X-D is higher than that predicted by *G*_*o*_*W*_*o*_@HF/BSE by 0.080 eV. Excitations of A_2_ and B_1_ symmetry are of DD and DA character and both ωB97X-D and M06-2X TDDFT kernels predict lower excitation energies than *G*_*o*_*W*_*o*_@HF/BSE by up to 0.3 eV. Singlet excitations of B_2_ symmetry are of AA character and *G*_*o*_*W*_*o*_@HF/BSE predicts these to be lower by around 0.25 eV.

**Table 13:** Mean Signed Difference in eV between *G*_*o*_*W*_*o*_@HF/BSE and TDDFT/ωB97X-D or TDDFT/M06-2X for the Average of the First Five Excitation Energies of Each Symmetry Type in the GS Geometry

	B97	M06	B97	M06
	singlets	singlets	triplets	triplets
A_1_	0.080	–0.012	–0.706	–0.872
A_2_	–0.088	–0.322	–0.278	–0.232
B_1_	–0.090	–0.326	–0.276	–0.216
B_2_	0.258	0.238	–0.696	–0.842

#### III.B.2 Emissive States in the CT Geometry

**Figure 4:**
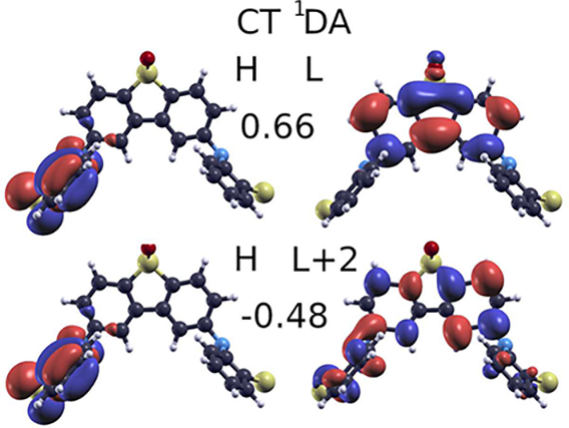
Localization of electrons and holes on D and A units for the CT geometry. Coefficients for the dominant singles configurations for the ^1^DA state are indicated.

When the molecular stucture is relaxed after a vertical excitation to the 1^1^DA state, the energy is lowered by 0.59 eV in *G*_*o*_*W*_*o*_@HF/BSE, by 0.60 eV in TDDFT/ωB97X-D and by 0.67 eV in TDDFT/M06-2X in the CT geometry. HF orbitals in this geometry and largest coefficients of the ^1^DA CT excited state are shown in Figure 4. Singlet transition energies to these levels listed in Table 8 change by 1.18, 0.95, and 1.07 eV, respectively. The difference arises because the HF and DFT ground state energies at the CT geometry increase. This makes the 1^1^DA state the lowest *singlet* state in the CT geometry, for each method. In contrast to the TDDFT methods, the ^1^DD level in *G*_*o*_*W*_*o*_@HF/BSE rises in the CT geometry. The lowest excited state in the CT geometry according to M06-2X is the ^3^DA state, which lies 0.31 eV below the ^3^DD state. The ωB97X-D functional predicts that the lowest excited state is the ^3^DD state, which lies 0.32 eV below the ^3^AA state and 0.34 eV below the ^3^DA state. *G*_*o*_*W*_*o*_@HF/BSE predicts the lowest excited state in the CT geometry to be the ^3^AA state with the ^3^DD state 0.17 eV higher. Figure 3 (top panel) shows that these triplet LE states lie well below the singlet states and the ^3^DA state. Peach and Tozer point out that triplet states with large electron–hole separations, as in these CT states, are less affected by the triplet instability.^79^ Indeed the singlet–triplet splitting for the ^1,3^DA states is just 25.4 meV (Table 10).

### III.C Optical Absorption Spectra

**Figure 5:**
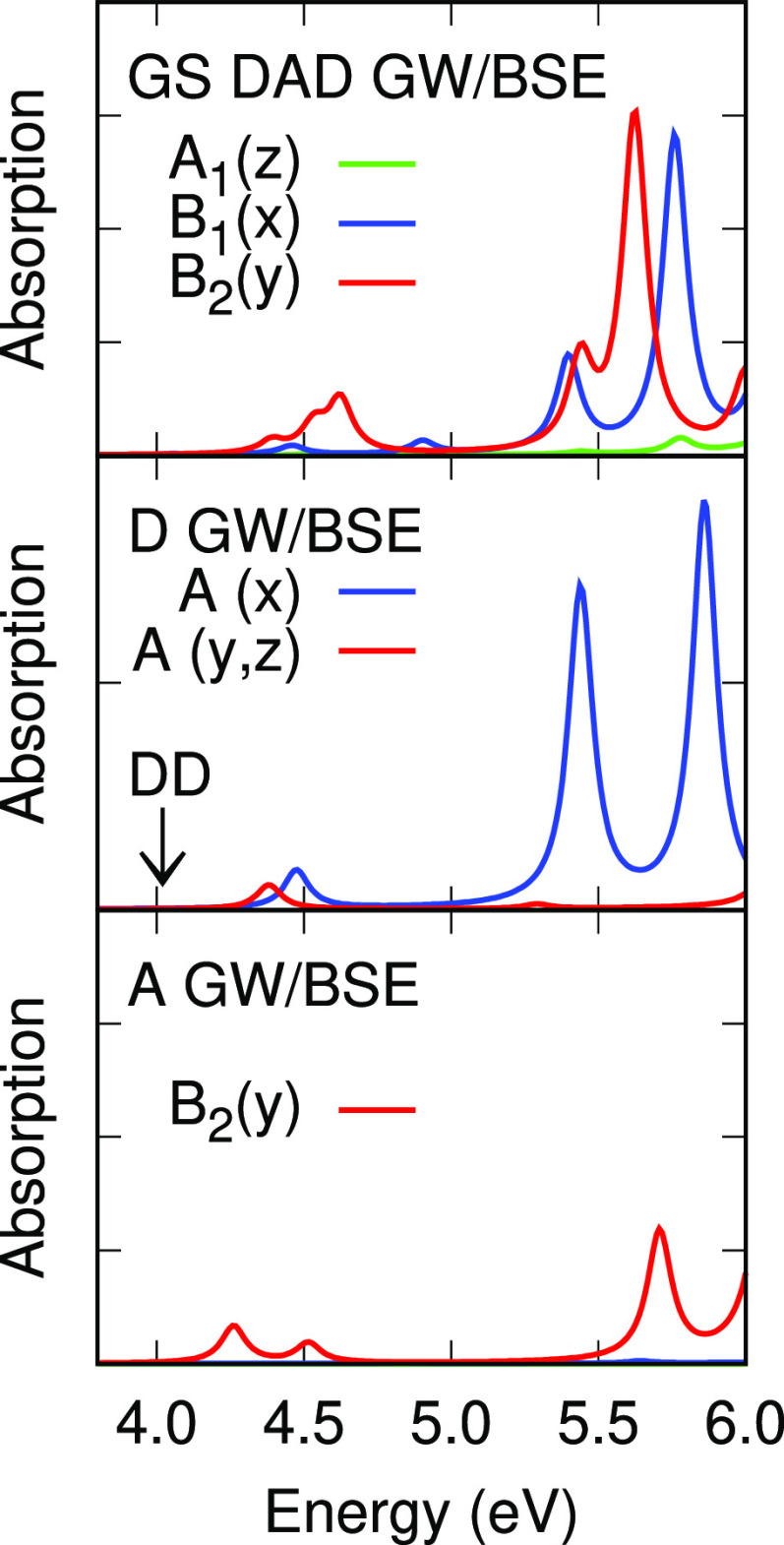
Vertical absorption spectra of the DBTO2 acceptor (A) and PTZ donor (D, scaled ×2) and DPTZ-DBTO2 emitter (DAD) from *G*_*o*_*W*_*o*_@HF/BSE. The position of a very weak donor transition is marked by DD.

**Figure 6:**
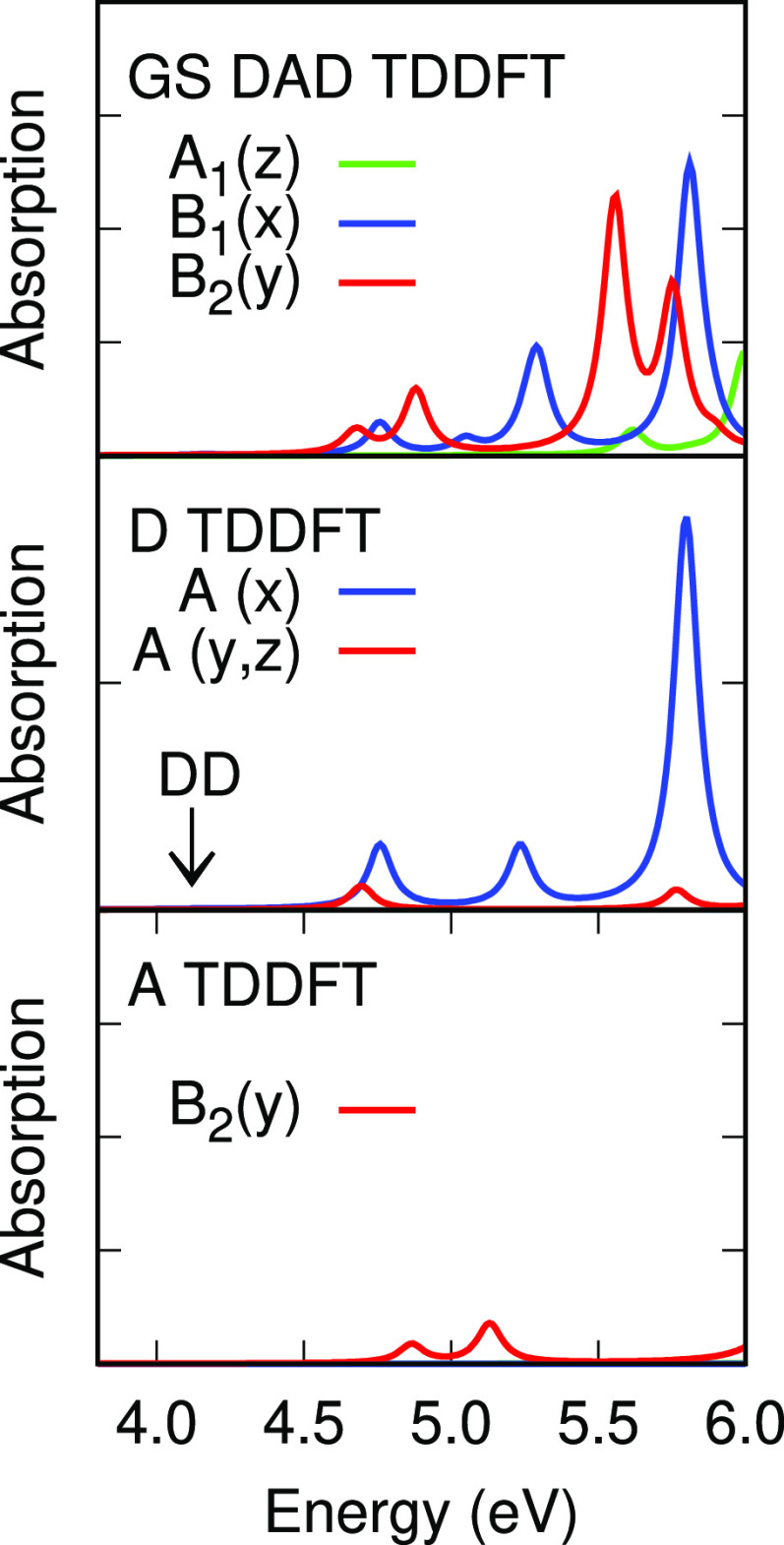
Vertical absorption spectra of the DBTO2 acceptor (A) and PTZ donor (D, scaled ×2) and DPTZ-DBTO2 emitter (DAD) from TDDFT/M06-2X. The position of a very weak donor transition is marked by DD.

Figures 5 and 6 show absorption spectra of the DBTO2 acceptor, PTZ donor, and DPTZ-DBTO2 TADF emitter from TDDFT/M06-2X and *G*_*o*_*W*_*o*_@HF/BSE. The acceptor lies in the *yz* plane and the *z* axis is the *C*_2*v*_ principal axis in both the A fragment and the DAD molecule. Transitions with the transition moment parallel to the A or DAD principal axis, *z*, and *x* and *y* axes belong to the A_1_, B_1_, and B_2_ irreducible representations of the *C*_2*v*_ point group, respectively. The isolated donor has *C*_*s*_(*x*) symmetry and its σ_*h*_ mirror plane is the *yz* plane, which it shares with the acceptor in the DAD molecule. Transitions with the transition moment parallel or perperdicular to the D σ_*h*_ mirror plane belong to the A′ and A″ irreducible representations, respectively.

#### III.C.1 Isolated Donor and Acceptor Optical Absorption

The PTZ donor shows six singlet transitions in the range 4 to 6 eV. Transition energies are given in Table 14 and optical absorption spectra from *G*_*o*_*W*_*o*_@HF/BSE and TDDFT/M06-2X methods are compared to absorption spectra for the DAD molecule in the GS geometry in Figures 5 and 6. Optical absorption spectra for D from *G*_*o*_*W*_*o*_@HF/BSE and TDDFT show good agreement, with the exception of the intensity of the second A″ transition, which is stronger in *G*_*o*_*W*_*o*_@HF/BSE. The donor H, H-1, and L MOs are even and the L and L+1 MOs are odd with respect to the σ_*h*_ mirror plane. The H/L+1 (A″) transition at 4.02 eV (marked DD in Figure 5) is very weak. It becomes the lowest ^1^DD transition in the DAD system, where it is weak also. The H/L (A′) transition at 4.37 eV has its transition moment perpendicular to the long *x* molecular axis and the H/L+2 (A″) transition at 4.47 eV has its transition moment along the *x* axis. The A″ transitions at 5.43 and 5.85 eV are mixtures of the H-1/L+1 and H/L+2 transitions. Most TDDFT transition energies for D are higher than *G*_*o*_*W*_*o*_@HF energies, by an average of 0.15 eV.

**Table 14:** Transition Energies and Irreducible Representations of Optically Active Transitions in the PTZ Donor (*C*_*s*_(*x*)) and DBTO2 Acceptor (*C*_2*v*_) Systems in eV

donor			acceptor		
irrep.	*G*_*o*_*W*_*o*_@HF	M06-2X	irrep.	*G*_*o*_*W*_*o*_@HF	M06-2X
A′	4.37	4.69	A1	4.82	5.41
A′	5.28	5.76	A1	5.64	6.37
A″	4.02	4.12	B2	4.25	4.86
A″	4.47	4.75	B2	4.51	5.13
A″	5.43	5.23	B2	5.70	6.18
A″	5.85	5.79			

The DBTO2 acceptor shows two weak and one stronger B_2_ singlet absorptions in the range 4 to 6.5 eV (Table 14 and Figures 5 and 6). The H and H-1 MOs are of *b*_2_ symmetry and the L and L+1 MOs are of *b*_1_ symmetry. Weak transitions at 4.25 and 4.51 eV in *G*_*o*_*W*_*o*_@HF/BSE are the H/L and H/L+1 transitions of B_2_ symmetry, with the transition moment along the long *y* axis. The H-1/L transition is at 5.70 eV and is also of B_2_ symmetry. All TDDFT singlet excitation energies for DBTO2 exceed *G*_*o*_*W*_*o*_@HF/BSE values, by an average of 0.57 eV. The absorption spectrum of DBTO2 measured in methylcyclohexane shows excitations at 3.9, 4.1, and 4.5 eV.^20^

#### III.C.2 Donor–Acceptor–Donor Optical Absorption and Emission

The DPTZ-DBTO2 emitter has 17 optically active transitions in the range 4 to 6 eV. The H to H-3 MOs are localized on D and the H-4 MO is localized on A (Supporting Information, Figures S1 and S2). L and L+1 are fully localized on A and L+2 is partly on D and A. On going from the GS geometry to the CT geometry, the HF L+4 orbital localized on D switches to L+3. The two largest coefficients in *G*_*o*_*W*_*o*_@HF/BSE or TDDFT eigenvectors for the excited states discussed in section III.B are compared in Tables 11 and 12.

**Figure 7:**
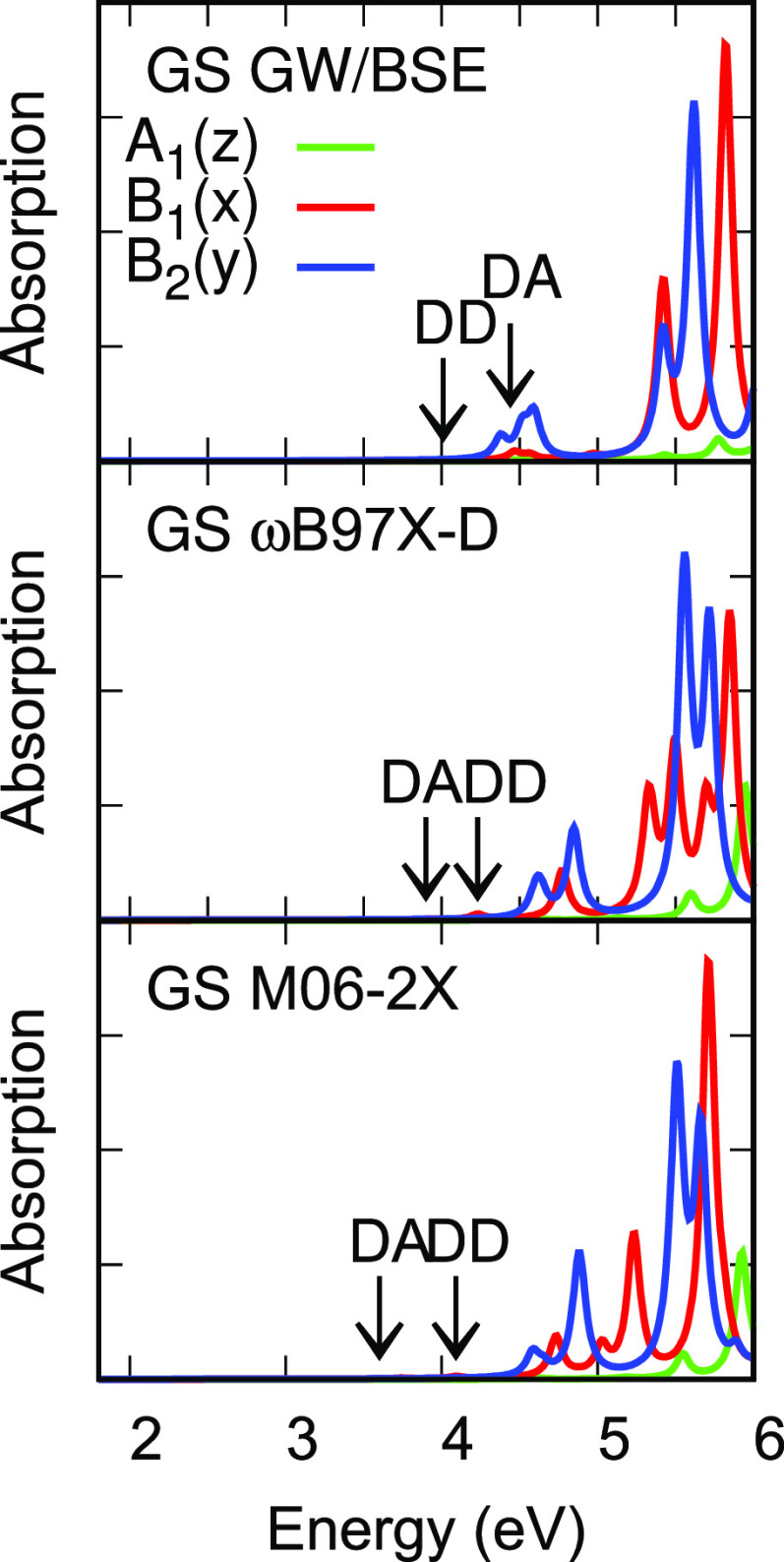
Vertical singlet absorption spectrum of DPTZ-DBTO2 in the GS geometry and emission spectra in the CT and LE geometries from *G*_*o*_*W*_*o*_@HF/BSE. Positions of DA CT and DD LE absorptions and emissions with very weak transitions are indicated by arrows.

**Figure 8:**
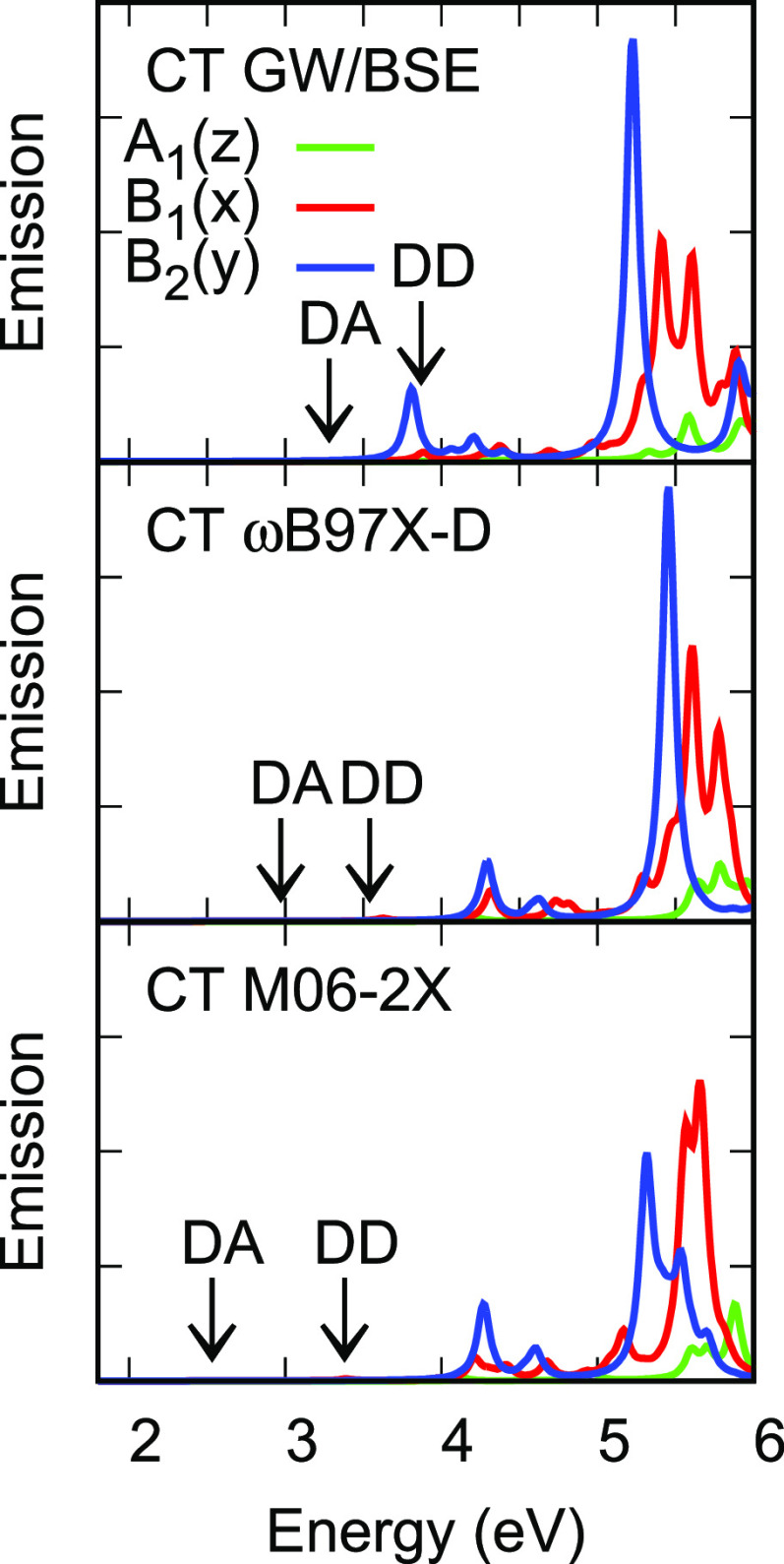
Vertical singlet absorption spectrum of DPTZ-DBTO2 in the GS geometry and emission spectra in the CT and LE geometries from TDDFT/M06-2X. Positions of DA CT and DD LE absorptions and emissions with very weak transitions are indicated by arrows.

Absorption spectra of the DAD system for the GS geometry and emission spectra for the CT geometry are shown in Figures 7 and 8. While appreciable fluorescence is expected only from the lowest excited state, comparison of transition matrix elements in this graphical form illustrates the extent to which predictions from *G*_*o*_*W*_*o*_@HF/BSE, TDDFT/ωB97X-D and TDDFT/M06-2X differ. Figure 2 shows single-particle levels for the GS and CT geometries. While the *C*_2*v*_ symmetry is broken in the CT geometry, levels shift to a limited extent and the main consequence of symmetry breaking for optical transitions is that orbitals which are delocalized over both donors become localized on one or other donor. DA transitions in the CT geometry are of ππ*character.

In the GS geometry the H-1 → L+4/H → L+2 A_2_ transition at 4.01 eV in *G*_*o*_*W*_*o*_@HF/BSE is dipole forbidden and the nearly degenerate H → L+4/H-1 → L+2 B_1_ transition is weak (Table 11 and top panel Figure 7). These are linear combinations of the very weak A″ transition found at 4.02 eV in the isolated donor. The lowest energy transitions in *G*_*o*_*W*_*o*_@HF/BSE at the GS geometry are therefore combinations of DD transitions, with coefficients of 0.57 and 0.50. The corresponding TDDFT/ωB97X-D and TDDFT/M06-2X DD B_1_ transitions at 4.23 and 4.09 eV are also weak and are also linear combinations of DD transitions (H → L+3/H-1 → L+4). with similar weights (Table 11 and top panel Figure 7). Note that there is a change in ordering of orbitals/orbital symmetries above L+2 for HF and DFT (see the Supporting Information, Figures S1 and S2). Thus, LE DD transition energies for the three methods lie within a range of 0.22 eV.

Etherington and co-workers^20^ reported optical absorption peaks in 2,8-DPTZ-DBTO2 in DCM at 3.71, 4.35, and 4.87 eV. Dias and co-workers^34^ reported the variation of the S_1_ B3LYP TDDFT excitation energy as a function of the dihedral angle containing the C–N bond connecting the D and A fragments of the 2,8-DPTZ-DBTO2 DAD system. They found that the S_1_ energy ranged from about 3.5 eV (in good agreement with the M06-2X value of 3.60 eV in Table 6) to about 2.4 eV as the dihedral angle ranged from 0 to 90°. Hence flexing of the molecule in solution may result in additional optical absorptions which have quite different energies and transition moments in the equilibrium geometry.

There are four DA transitions in *G*_*o*_*W*_*o*_@HF/BSE at 4.45, 4.46, 4.52, and 4.56 eV in the GS geometry. These are two pairs of B_1_ and A_2_ transitions between H and H-1 and L and L+1 (Tables 6 and 11). The lower B_1_ H → L/H → L+1 B_1_^1^DA transition predicted by *G*_*o*_*W*_*o*_@HF/BSE at 4.46 eV is very weak and the second B_1_ transition at 4.56 eV can be seen in the top panel of Figure 7. The excited state dipole moments of the first two states with DA character in *G*_*o*_*W*_*o*_@HF/BSE are 11.85 and 9.88 D and the latter two states with DA character have larger dipole moments (14.83 and 16.21 D).

The corresponding B_1_ and A_2_ excited state transition energies in TDDFT/ωB97X-D are found at 3.90, 3.91, 3.91, and 3.95 eV and in TDDFT/M06-2X they occur at 3.60, 3.61, 3.74, and 3.75 eV, and all are weak (middle panel Figure 7). In contrast to *G*_*o*_*W*_*o*_@HF/BSE, the excited state dipole moments of the first two states in TDDFT/ωB97X-D and TDDFT/M06-2X are under 10 D and the latter pairs of states have lower dipole moments again (Table 6). Participation of D and A frontier orbitals in low energy transitions does not therefore guarantee a state of true CT character and *G*_*o*_*W*_*o*_@HF/BSE and TDDFT methods in this work predict somewhat different characters for these states. Excited state dipole moments for these states depend on the linear combinations of transitions involved as well as their relative phases. States labeled as DD or DA above belong to the same B_1_ and A_2_ irreducible representation and mixing of DD and DA transitions determines the excited state character (LE or CT). *G*_*o*_*W*_*o*_@HF/BSE predicts the LE ^1^DD transition to lie below the ^1^DA transitions while the TDDFT methods predict the ^1^DA transition energies to be lower.

The lowest energy states with B_2_ symmetry are mainly AA in character with significant weights of the H-4/L and H-4/L+1 transitions (Table 11) and the transition moment polarized along the *y* axis. Excitation energies rise from 4.38 to 4.65 eV. These LE AA excitation energies have a limited dependence on the method, as was found for LE DD states.

Inspection of GS singlet excitation energies (Table 6), comparison of excitation energies for each irreducible representation and comparison of wave function amplitudes show that there are clear trends in these quantities on going from *G*_*o*_*W*_*o*_@HF/BSE, via TDDFT/ωB97X-D to TDDFT/M06-2X. The GS 1^1^DA transition is a linear combination of H/L and H/L+1 transitions (Table 11). The relative weight of each transition varies continuously from *G*_*o*_*W*_*o*_@HF/BSE to TDDFT/M06-2X, excited state dipole moments are roughly the same (9.88, 7.92, and 9.12 D, respectively), and the excitation energy reduces sharply going from *G*_*o*_*W*_*o*_@HF/BSE to TDDFT/M06-2X (4.46, 3.90, and 3.60 eV, respectively). Absorption spectra for the GS geometry from these three methods in Figure 7 show good agreement for both *x* and *y* incident light polarization.

#### III.C.3 Singlet–Triplet Splitting

Excitation energies, excited state dipole moments, and singlet–triplet splittings predicted by each method are given in Table 10. Singlet–triplet splittings for the first two excited states of DA character are given for *G*_*o*_*W*_*o*_@HF/BSE and TDDFT/ωB97X-D, and the first four are given for TDDFT/M06-2X since four states of clear DA character can be identified in that case. In the GS geometry the splittings of the first two DA states reduce on going from *G*_*o*_*W*_*o*_@HF/BSE to TDDFT/M06-2X. *G*_*o*_*W*_*o*_@HF/BSE predicts splittings for these states of nearly 300 meV. This reduces to 17.9 meV in TDDFT/ωB97X-D and to around 5 meV in TDDFT/M06-2X. In that case it was possible to obtain splittings for the third and fourth states of DA character and these are around −40 meV. DA states are linear combinations of transitions from the H and H-1 levels to the L and L+1 levels. The change in signs of the splittings in the third and fourth levels is expected to be due to changes in phases of the excited state wave functions for those cases. Sizes of excited state dipole moments also change steadily on going from *G*_*o*_*W*_*o*_@HF/BSE to TDDFT/M06-2X. The dipole moment in the 1^1^DA state changes from 11.85 D in *G*_*o*_*W*_*o*_@HF/BSE to 6.52 D in TDDFT/M06-2X.

The singlet–triplet splitting for the CT geometry is the more important one, from a TADF point of view. Triplet states in this geometry may undergo rISC and delayed fluorescence, provided the splitting is of order *kT* or less. The large splittings predicted by *G*_*o*_*W*_*o*_@HF/BSE in the GS geometry are much reduced, to 25.4 and 9.3 meV for the first two states of DA character. TDDFT/ωB97X-D predicts splittings of −61.1 and 6.1 meV in this geometry and TDDFT/M06-2X predicts larger splittings for the first two DA states (50.2 and 23.0 meV) and negative splittings (−23.7 and −23.2 meV) for the next two DA states. Magnitudes of excited state dipole moments are larger in the CT geometry and show the same magnitude trend from *G*_*o*_*W*_*o*_@HF/BSE to TDDFT/M06-2X as in the GS geometry. The increase in magnitude arises as holes localize in one donor fragment in the CT geometry, leading to dipole moment components in both the *y* and *z* directions, whereas there is only a *z* component in the GS geometry.

Peach and Tozer^79^ found that underestimation of singlet and triplet excitation energies by the PBE GGA functional, compared to third order coupled cluster values, was corrected using a range-separated CAM-B3LYP functional, for both LE and CT excitations. They also note that the triplet instability problem affects LE states to a much greater extent than CT states. Hence it is not surprising that *G*_*o*_*W*_*o*_@HF/BSE predicts singlet–triplet splittings in the expected range for the CT geometry. The larger splittings predicted by *G*_*o*_*W*_*o*_@HF/BSE in the GS geometry may arise from greater mixing of DA and DD configurations. In the GS geometry the 1^1^DD, 1^1^DA, and 1^3^DA states are close in energy while the 1^3^DD state lies lower (Figure 3 top panel). Since each of these states is of the same symmetry, greater mixing of the DA and DD configurations may occur for singlets compared to corresponding triplet states and lead to the large singlet–triplet splitting in the GS geometry. In the CT geometry, the ^1,3^DA states are further from the 1^1^DD state, resulting in less mixing and a 10 times smaller singlet–triplet splitting. Inspection of BSE excited state wave functions for the GS and CT geometries in Tables 11 and 12 shows that there are much larger changes in coefficients of H/L and H/L+1 transitions on going from the GS to CT geometry for BSE compared to the TDDFT methods.

## IV Discussion

The main variations in predictions of the *G*_*o*_*W*_*o*_@HF/BSE and TDDFT methods for excited states of DPTZ-DBTO2 are in the predicted single-particle energy gaps, in the relative stabilities of local exciton singlets and triplets, and in the relative stabilities of local exciton and charge transfer states. Both methods agree that the singlet–triplet splittings of charge transfer DA states are small (of the order of a few meV). These results are discussed in this section as well as changes in excitation energies in solution or condensed phases.

### IV.A DD versus DA Excitations

Figure 2 shows single-particle levels for the DAD system in the GS and CT geometries and Table 2 gives their single-particle gaps, i.e., the smallest gaps of DD, AA, and DA levels and the difference in DD and DA gaps. *G*_*o*_*W*_*o*_@HF gaps exceed M06-2X gaps by over 2 eV, except for the DA gap in the CT geometry. ωB97X-D gaps lie between those extremes and closer to *G*_*o*_*W*_*o*_@HF gaps. The difference in DD and DA gaps is nearly constant for each method and rises by 0.2 to 0.3 eV on going from the GS to CT geometry. Figures 7 and 8 show that differences in the lowest DD and DA excitation energies also increase with the change in geometry.

Low level coupled cluster calculations such as CC2, which include double excitations perturbatively, would yield additional insights into the relative accuracy of these TDDFT and *G*_*o*_*W*_*o*_@HF/BSE methods. In particular, relative weights of *T*_1_/*T*_2_ amplitudes in the triplet states could reveal the origin of the triplet instability of the *G*_*o*_*W*_*o*_@HF/BSE method in LE states.

Table 14 compares transition energies for D and A systems in their equilibrium geometries and it is perhaps surprising to find that the first five (six) excitation energies predicted for the A (D) systems by TDDFT/M06-2X are 0.15 (0.57) eV higher than predicted by *G*_*o*_*W*_*o*_@HF/BSE. This observation also holds in DAD where LE excitations of B_2_ symmetry (mainly AA transitions) and A_1_ symmetry are predicted by TDDFT/M06-2X to be 0.15 and 0.02 eV higher in energy, compared to *G*_*o*_*W*_*o*_@HF/BSE (Supporting Information, Table S12, columns 1 and 7). On the other hand, excitations of B_1_ symmetry (mainly DA transitions) are predicted by TDDFT/M06-2X to be −0.45 eV lower on average. Even though differences in DD and DA gaps according to either method are roughly the same, TDDFT/M06-2X predicts relatively lower DA transition energies compared to *G*_*o*_*W*_*o*_@HF/BSE by around 0.5 eV.

Underestimation of CT excitation energies by TDDFT methods and a remedy of adding a fraction of exact exchange were highlighted some time ago.^35^ The TDDFT f_*xc*_ kernel which replaces the Coulombic electron–hole attraction is short-ranged in a nonhybrid density functional, resulting in predicted CT excitation energies which approximate single-particle excitation energies.^35^ A heuristic explanation for the observation that TDDFT local exciton energies are larger than *G*_*o*_*W*_*o*_@HF/BSE energies while CT energies are smaller is that screening of electron–hole attraction in *G*_*o*_*W*_*o*_@HF/BSE depends on the distance between electron and hole in a manner similar to electron–hole attraction in a range-separated TDDFT approach. In the latter, it is strongly screened at short-range and essentially unscreened beyond a cutoff distance around 8a_*o*_.^80^ On the other hand, in TDDFT/M06-2X the electron–hole attraction is scaled by 54%, independent of electron–hole separation. Thus, the latter will underscreen at short-range, leading to more HF-like overestimates of excitation energies in local excitons and overscreen at long-range, leading to relative underestimation of CT excitons.

### IV.B Singlet–Triplet Exchange Splitting

rISC in TADF emitters is a relatively slow process and therefore the relevant molecular geometry in which to consider the rISC rate is the CT geometry. Inspection of Table 10 shows that singlet–triplet splittings vary markedly on going from the GS to CT geometry. However, many calculations of these splittings in TADF molecules are performed using the GS geometry and frequently the exchange integral is used in place of a TDDFT or BSE calculation. Variations in singlet–triplet splitting arise because the LE or CT character of low energy excited states can vary markedly with geometry. For example, the main configuration of the 1^1^DD state in the GS geometry predicted by *G*_*o*_*W*_*o*_@HF/BSE (top row of Table 11) changes on going to the CT geometry (top row of Table 12) and the singlet–triplet splitting changes from around 300 meV to less than 30 meV. The splittings of the 1^1,3^DA and 2^1,3^DA states in the GS geometry according to TDDFT/ωB97X-D are both around 18 meV (Table 10) but the splitting of the 1^1,3^DA state becomes −61.1 meV in the CT geometry. This may arise because the 1^3^AA state (2.99 eV) is close in energy to the 1^3^DA state (3.01 eV) only in the CT geometry. Hence these splittings must be calculated in the relevant CT geometry and predictions will depend on TDDFT f_*xc*_ kernel or screened interaction in BSE to some extent. Except for the case just mentioned, singlet–triplet splittigs are positive for the first two pairs of ^1,3^DA states. Negative splittings occur for the third and fourth pairs of states, presumably because of orthogonality to the lower pairs of these states. In contrast to triplet energies of LE states which are subject to triplet instability (Table 13), singlet–triplet splittings in *G*_*o*_*W*_*o*_@HF/BSE are in reasonable agreement with values from TDDFT/M06-2X.

### IV.C Excitation Energies in Various Media

Single particle energy levels and optical excitation energies presented here are for isolated molecules. These values are larger than experimental values obtained in solution or in the condensed phase. Polarization in the molecular environment reduces the H-L gap in solution and optical transition energies. Tables 4 and 5 report differences in C-PCM energies for neutral and ionized D, A and DAD systems and predicted shifts in H and L energy levels derived from C-PCM calculations. The simple physical model on which these shifts are based (eq 8) is a neutral molecule or ion in a cavity surrounded by a continuum dielectric. Multipole moments of the molecule or ion polarize the continuum resulting in induced charges on the surface of the cavity which contribute to the electrostatic energy of the molecule or ion. Interaction strength depends on the order of the moment^81^ and for the D, A and DAD systems, C-PCM energies for the neutral systems are three or four times smaller than the ionized systems.

Combined estimated shifts in IP and EA in a CHX solvent (Table 4) amount to 1.5 eV for the CT state geometry. When a 1.5 eV shift in *G*_*o*_*W*_*o*_@HF eigenvalues in RPA and BSE A matrices (Table 1) is made, the ^1^DA excitation energy is reduced by a nearly rigid shift of 1.55 eV. While emission spectra for DPTZ-DBTO2 do extend down to this energy range,^34^ it is difficult to quantify the accuracy of this prediction.

OLED emitters are examples of amorphous^5^ or crystalline^20^ luminescent organic condensed phases. In many cases these emitters are embedded in a similar host matrix with a larger band gap, to ensure emission via the emitter. Excited states in organic molecular crystals such as the polyacenes have been extensively investigated and the local exciton and intermolecular CT character of their excitations is well-known.^82^ Molecules which are conjugated throughout, such as polyacenes, only have the possibility of intermolecular CT excitons, whereas TADF molecules in the condensed phase have the additional possibility of intramolecular CT states—the ^1,3^DA states in this work. TADF molecules in either amorphous or crystalline condensed phases have a much different dielectric background to the gas or solution phases and prediction of the impact of potential intermolecular versus intramolecular CT states on luminescence is unknown. For example, does recombination occur via intermolecular as well as intramolecular CT states? The former must be formed as intermediate states when an electron and hole are in the proximity of the emitter. Condensed phase simulations are necessary in order to answer these questions.

Work to determine how best to incorporate effects of the dielectric background on light emission is in progress. Sun and co-workers^83^ used an optimally tuned range-separated TDDFT approach to account for the differences in molecular H-L gaps and band gaps of the molecular crystals for a series of conjugated organic molecules. They found that the tuning needed to be carried out in the presence of the C-PCM response and that the predicted reduction in energy gap varied linearly with the difference in polarization energy, which is proportional to (1 – 1/ε), as expected from eq 8.

Northey et al.^23^ simulated the solvated ^1^CT excited state of PTZ-DBTO2 (DA) using both C-PCM and explicit solvent molecules. In the ground state geometry they found a small increase in absorption energy (up to 0.1 eV going from vacuum to a polar solvent, EtOH). However, in a state-specific TDDFT/M06-2X calculation, the excitation energy reduced from 2.65 eV in vacuum (similar to the 2.53 eV in vacuum energy reported in Table 8 for the DAD system) to 2.08 eV in CHX, 1.45 eV in DCM, and 1.30 eV in EtOH. In this case, the cost of creating the large excited state dipole moment in the ^1^CT state is reduced by screening by the PCM and changes in excitation energy are of a similar magnitude to the value of 1.55 eV noted above.

The Exciton code used in this work has been applied to a molecular crystal^84^ using a density fitting method based on Gaussian orbitals.^54^ A density fitting method combined with a local orbital basis in the Exciton code will permit molecular crystals with larger molecules to be treated using methods which incorporate the electron–hole attraction in the excited state Hamiltonian and therefore help to determine the nature of TADF molecules in their environment in an OLED device.

## V Conclusions

In this work, we presented a comparison of single-particle and excited state energy levels and optical transitions of the DPTZ-DBTO2 molecule predicted by the M06-2X and ωB97X-D functionals in TDDFT and *G*_*o*_*W*_*o*_@HF/BSE, in the gas phase. Shifts in H and L levels of the molecule were estimated using a conductor-like PCM model. Calculations were performed in the ground state equilibrium (GS) geometry and a ^1^DA charge transfer excited state (CT) geometry. Relative stabilities of specific excited states depend on the geometry and method used. The lowest singlet found by *G*_*o*_*W*_*o*_@HF/BSE is a LE ^1^DD state and the CT ^1^DA state is predicted to be 0.44 eV higher using a TZVP basis. In contrast the lowest singlet found by TDDFT/ωB97X-D and TDDFT/M06-2X methods is the CT ^1^DA state and the LE ^1^DD state is 0.33 (0.49) eV higher in energy. This reflects a systematic trend found in excited states of the isolated donor (D) and acceptor (A) molecules as well as the DAD molecule. TDDFT/M06-2X typically predicts local exciton states to be 0.1 to 0.2 eV higher in energy than *G*_*o*_*W*_*o*_@HF/BSE, while it predicts charge transfer states to be 0.4 eV lower in energy. Predictions of the range-separated TDDFT/ωB97X-D method typically lie between the other methods. This accounts for the reversal in order of the LE ^1^DD and CT ^1^DA states by the methods in the GS geometry. Oscillator strengths for optical transitions in the GS and CT geometries from all methods used agree reasonably well, and it is possible to identify corresponding transitions in each method. Oscillator strengths for the lowest singlet excited state in each geometry and method are very low.

In the CT geometry, the 1^1^DA and 1^1^DD states reverse in order and all methods predict the 1^1^DA state to be the lowest excited state. TDDFT/M06-2X predicts this state at 2.53 eV, TDDFT/ωB97X-D at 2.95 eV and *G*_*o*_*W*_*o*_@HF/BSE at 3.28 eV. Both *G*_*o*_*W*_*o*_@HF and DFT/ωB97X-D predict the IP to lie below the value predicted by a ΔSCF calculation and the EA to be smaller than the ΔSCF values while DFT/M06-2X predicts the IP to lie above the ΔSCF value and the EA to be larger than the ΔSCF value. It is therefore likely that TDDFT/M06-2X underestimates the 1^1^DA excitation and TDDFT/ωB97X-D and *G*_*o*_*W*_*o*_@HF/BSE overestimate it. Obtaining the correct single-particle gap in the SCF state used for a linear response calculation may be crucial in predicting the best excitation energies and these can be judged using the ΔSCF value.^80^ It is recommended that singlet–triplet splittings in TADF emitters be calculated in the relevant CT geometry rather than the vertical splitting as predicted values can differ significantly between geometries.

Predictions of singlet–triplet splitting for ^1,3^DA and ^1,3^AA states by either method are generally in agreement. However, anomalies exist which may be explained by mixing of either the singlet or triplet with other states, which can alter the predicted splitting significantly. 1^1,3^DA splittings, which are critical for TADF applications, are predicted to be 25.4, −61.1, and 50.2 meV, according to *G*_*o*_*W*_*o*_@HF/BSE and TDDFT/ωB97X-D and TDDFT/M06-2X, respectively.

## Data Availability

The data that support the findings of this study are available from the corresponding author upon reasonable request. The Exciton code used in this work is available from CHP under a Mozilla Public License 2.0 on reasonable request, via email to Charles.Patterson@tcd.ie.

## Supporting Information Available

Bond lengths to heteroatoms in DPTZ-DBTO2, isosurface plots of HF and DFT/M06-2X molecular orbitals used in *G*_*o*_*W*_*o*_@HF/BSE and TDDFT/M06-2X calculations, and molecular geometries in the GS and CT excited state as well as excited state energies for singlet and triplet states in GS geometries from various methods used in the work (PDF)
