# Supplementary material for: TDDFT versus GW/BSE Methods for Prediction of Light Absorption and Emission in a TADF Emitter
Source: J Phys Chem A. 2022 Dec 14;126(51):9627–43. doi: 10.1021/acs.jpca.2c06403 (PMC9806837; doi:10.1021/acs.jpca.2c06403)
Supplement: Supplementary file 1 — jp2c06403_si_001.pdf [file jp2c06403_si_001.pdf]

Supporting Information for TDDFT versus GW/BSE Methods for Prediction of Light Absorption and Emission in a TADF Emitter

D. Chaudhuri and C. H. Patterson*

School of Physics, Trinity College Dublin, Dublin 2, Ireland

E-mail: chaudhud@tcd.ie

This document contains: bond lengths to heteroatoms in DPTZ-DBTO2, isosurface plots of HF and DFT/M06-2X molecular orbitals used in G_oW_o@HF/BSE and TDDFT/M06-2X calculations, molecular coordinates in the GS and CT excited states as well as excited state energies for singlet and triplet states in GS geometries from various methods used in the work.

Comparison of bond lengths in the ground state and CT excited state predicted by HF, B3LYP, B3LYP+D3-BJ, TPSSh + D3-BJ, CIS, TD-B3LYP and TD-ωB97X-D functionals

Differences in bond lengths in excited states of organic molecules predicted by TDDFT are larger for carbon bonds to heteroatoms.[1] We illustrate the impact of ground and excited state optimization method on molecular structure in Supporting Information Table S1 using bond lengths to heteroatoms. Optimizing the ground state geometry using the HF and B3LYP methods results in S=O bond lengths which differ by 3.1 pm, however, addition of the D3-BJ functional to B3LYP reduces this to 1.4 pm. The two distinct C-S bond lengths in DPTZ-DBTO2 differ by 2.2 and 0.4 pm using these methods and these change to 1.0 and -0.5 pm with the addition of the B3-DJ functional. Differences in bond lengths predicted by B3LYP + D3-BJ versus TPSSh + D3-BJ are 0.5 pm or less. Turning to CT excited state bond lengths, the S=O bond length predicted by CIS is shorter than the TD-B3LYP S=O bond length by 2.9 pm. The ωB97X-D functional (without the D3-BJ dispersion correction predicts it to be 0.1 pm shorter than the TD-B3LYP S=O bond length. CIS predicts the C-S bonds to be between 0.2 and 4.0 pm shorter than TD-B3LYP, while ωB97X-D predicts them to be close to B3LYP-CT. The main change in the molecular geometry in the CT state is planarization of the PTZ donor which acquires most of the hole density in the CT ^{1}DA excited state.

Bond Lengths from HF, CIS and DFT Geometry Optimization Methods with various Basis Sets and Functionals for GS and CT DPTZ-DBTO2

Table S1: Bond Lengths to Heteroatoms in Å for DPTZ-DBTO2 in GS and CT geometries. Geometries obtained using HF, CIS, (TD)DFT-B3LYP, DFT-TPSSh and TDDFT-ωB97X-D functionals with KTZVP or cc-pVDZ basis sets.

Bond	RHF-GS[1]	B3LYP-GS[2]	B3LYP-GS[3]	TPSSh-GS[4]	CIS-CT[5]	B3LYP-CT[6]	ωB97X-D-CT[7]
S1-O4	1.439	1.470	1.453	1.456	1.451	1.480	1.479
S1-O5	1.439	1.470	1.453	1.456	1.451	1.480	1.479
S1-C8	1.780	1.802	1.790	1.785	1.787	1.785	1.774
S1-C9	1.780	1.802	1.790	1.785	1.742	1.782	1.792
S2-C26	1.776	1.780	1.771	1.768	1.775	1.781	1.776
S2-C27	1.776	1.780	1.771	1.768	1.775	1.781	1.776
S3-C28	1.776	1.780	1.771	1.768	1.741	1.746	1.738
S3-C29	1.776	1.780	1.771	1.768	1.741	1.746	1.738
N6-C16	1.427	1.435	1.433	1.434	1.432	1.445	1.436
N6-C30	1.418	1.424	1.421	1.420	1.418	1.419	1.413
N6-C31	1.418	1.424	1.421	1.420	1.418	1.419	1.413
N7-C17	1.427	1.435	1.433	1.434	1.410	1.472	1.440
N7-C32	1.418	1.424	1.421	1.420	1.388	1.391	1.387
N7-C33	1.418	1.424	1.421	1.420	1.388	1.391	1.387

[1] GS/RHF/KTZVP
[2] GS/B3LYP/cc-pVDZ
[3] GS/B3LYP+D3-BJ/cc-pVDZ
[4] GS/TPSSh+D3-BJ/cc-pVDZ
[5] CT/CIS/KTZVP
[6] CT/TD-B3LYP/cc-pVDZ
[7] CT/TD-ωB97X-D/cc-pVDZ

HF and M06-2X Kohn-Sham Molecular Orbitals

Occupied and virtual HF and M06-2X Kohn-Sham orbitals are shown in Figs. 1 and 2. In the C_{2v} GS geometry the nearly degenerate H and H-1 and H-2 and H-3 orbitals are shown in pairs. These localize on the donor (D) fragments and occur as linear combinations of D π states. The highest occupied acceptor (A) state is the H-4 level. In the GS geometry the first three virtual levels are localized on the A fragment and the L+2 level is partly delocalized onto the D fragments. The L+3 and L+4 levels are localized on the D fragments. On going to the CT geometry the L+3 and L+4 levels switch order and the H and H-1 and H-2 and H-3 levels localize on one D fragment or the other.

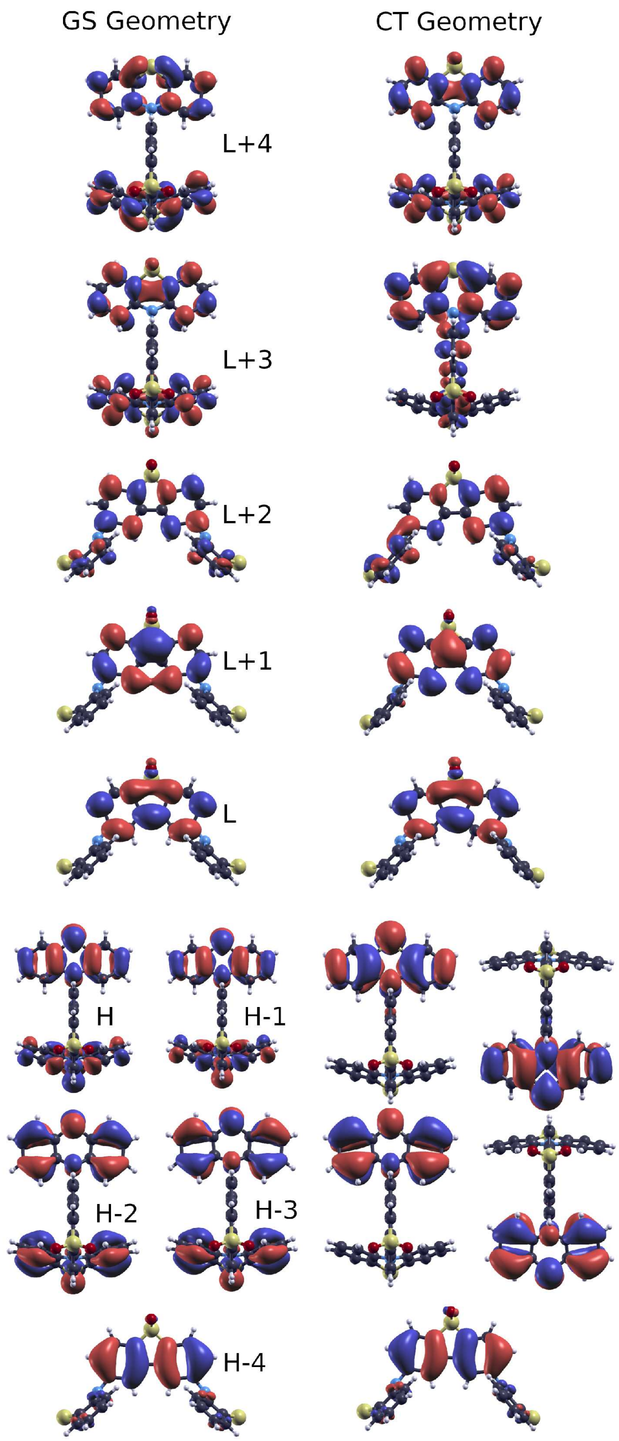

Figure S1: HF molecular orbitals for the GS and CT geometries. Orbital labels shown for the GS geometry apply to orbitals in equivalent positions in the CT Geometry column.

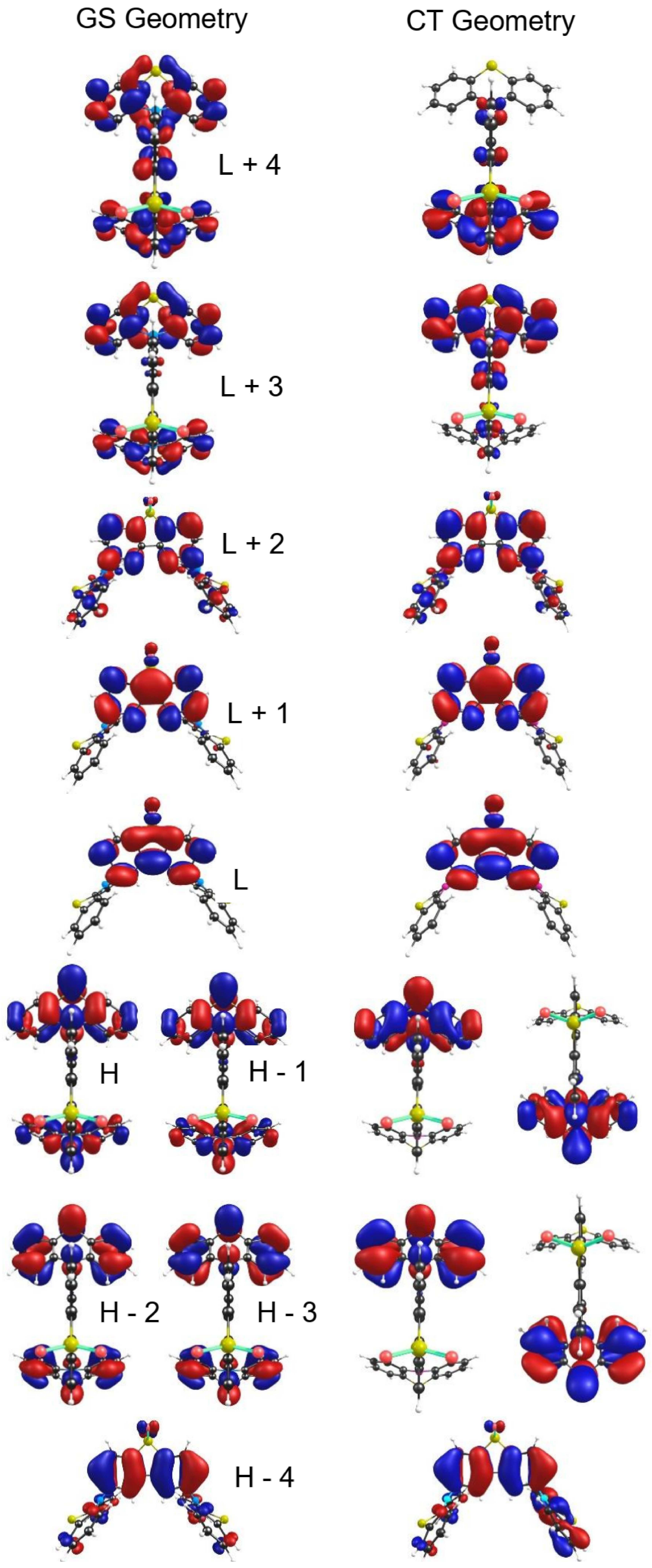

Figure S2: DFT/M06-2X molecular orbitals for the GS and CT geometry. Orbital labels shown for the GS geometry apply to orbitals in equivalent positions in the CT Geometry column.

HF/CIS/KTZVP and DFT/TDDFT-TDA/cc-pVDZ GS and CT geometries for DPTZ-DBTO2.

Table S2: 23 symmetry unique coordinates for DPTZ-DBTO2 molecule in HF/KTZVP C_{2v} GS geometry.

16	−0.0000000000	0.0000000000	−0.1668140857
16	0.0000000000	6.2492111158	−6.4923984020
8	1.2368152630	0.0000000000	0.5696670815
7	−0.0000000000	3.8539730688	−4.6764553754
6	0.0000000000	1.2828762297	−1.4066240419
6	0.0000000000	0.7403105775	−2.6807619765
6	−0.0000000000	1.5846535094	−3.7677952775
1	−0.0000000000	1.2015432817	−4.7702402314
6	−0.0000000000	2.9577097371	−3.5658201492
6	0.0000000000	3.4783013358	−2.2855637817
1	0.0000000000	4.5430517845	−2.1567257467
6	0.0000000000	2.6350145757	−1.1835226016
1	0.0000000000	3.0335402101	−0.1868382312
6	1.3452695636	5.1122448793	−6.2673939777
6	1.2307976982	4.0735456177	−5.3463281399
6	2.3578586408	3.3009861413	−5.0939753523
1	2.3263969298	2.5127346865	−4.3706298578
6	3.5487758226	3.5456727085	−5.7528117297
1	4.4037591218	2.9364722145	−5.5244559480
6	3.6391226299	4.5479727407	−6.6948990225
1	4.5575740158	4.7295601042	−7.2200156937
6	2.5245688209	5.3223182265	−6.9534054349
1	2.5703505070	6.1136969709	−7.6791541744

Table S3: 23 symmetry unique atomic coordinates for DPTZ-DBTO2 molecule in DFT/B3LYP/cc-pVDZ C_{2v} GS geometry.

16	0.0000000000	0.0000000000	−0.1705437812
16	−0.0000000000	6.4242545880	−6.3943870752
8	1.2691503674	0.0000000000	0.5705792434
7	0.0000000000	3.8975190689	−4.7161344731
6	0.0000000000	1.2922859048	−1.4272631835
6	0.0000000000	0.7393510310	−2.7159625089
6	0.0000000000	1.5990506304	−3.8152197815
1	0.0000000000	1.2132540182	−4.8306018929
6	0.0000000000	2.9860736910	−3.6072816966
6	0.0000000000	3.5107594794	−2.3088932104
1	0.0000000000	4.5885108678	−2.1810862508
6	0.0000000000	2.6595704583	−1.1992062316
1	0.0000000000	3.0602610729	−0.1899248612
6	1.3546574164	5.2814434686	−6.2231574451
6	1.2412107461	4.1768656392	−5.3559173782
6	2.3817490033	3.3883782600	−5.1375157513
1	2.3356816851	2.5417813338	−4.4629769497
6	3.5917065174	3.6891127493	−5.7655330727
1	4.4586781390	3.0658196150	−5.5635092808
6	3.6852482777	4.7626305354	−6.6478260439
1	4.6217862159	4.9917726639	−7.1478785125
6	2.5564638200	5.5490275867	−6.8807746026
1	2.6048801128	6.3951359990	−7.5609695566

Table S4: 23 symmetry unique atomic coordinates for DPTZ-DBTO2 molecule in DFT/B3LYP+D3-BJ/cc-pPVDZ C_{2v} GS geometry.

16	−0.0000000000	0.0000000000	−0.1581729985
16	−0.0000000000	6.2224339113	−6.6442838189
8	1.2591740087	0.0000000000	0.5672732162
7	−0.0000000000	3.8910830766	−4.6915993065
6	−0.0000000000	1.2851422805	−1.4053815017
6	−0.0000000000	0.7383191631	−2.6999697352
6	−0.0000000000	1.5986337150	−3.7974506595
1	−0.0000000000	1.2152966659	−4.8189686198
6	−0.0000000000	2.9841529226	−3.5813809630
6	−0.0000000000	3.5086432294	−2.2843043909
1	−0.0000000000	4.5918342292	−2.1588583045
6	−0.0000000000	2.6537165216	−1.1749131788
1	−0.0000000000	3.0506093258	−0.1586324123
6	1.3494226199	5.1162925334	−6.3363266423
6	1.2397509304	4.1138065997	−5.3497842812
6	2.3865593655	3.3682152198	−5.0304924824
1	2.3390300577	2.6050954511	−4.2559787758
6	3.6006673683	3.6055101827	−5.6809082166
1	4.4761457464	3.0166963584	−5.3998864529
6	3.6917208791	4.5719304711	−6.6815540419
1	4.6346575317	4.7519021623	−7.2006866124
6	2.5569843724	5.3173713438	−7.0108155950
1	2.6030939771	6.0849062842	−7.7861743121

Table S5: 23 symmetry unique atomic coordinates for DPTZ-DBTO2 molecule in DFT/TPSSh+D3-BJ/cc-pVDZ C_{2v} GS geometry.

16	−0.0000000000	0.0000000000	−0.1632103318
16	0.0000000000	6.2288228317	−6.6173771446
8	1.2624297905	0.0000000000	0.5634765349
7	0.0000000000	3.9012813683	−4.6807829566
6	0.0000000000	1.2820368051	−1.4061679392
6	0.0000000000	0.7375384684	−2.7024428486
6	0.0000000000	1.6016449734	−3.7993939277
1	0.0000000000	1.2239947005	−4.8229589791
6	−0.0000000000	2.9867014606	−3.5756007306
6	0.0000000000	3.5097981843	−2.2768849303
1	0.0000000000	4.5924305625	−2.1494668540
6	−0.0000000000	2.6514904170	−1.1703322804
1	−0.0000000000	3.0445145377	−0.1531382580
6	1.3458052134	5.1187826235	−6.3300093845
6	1.2380536310	4.1151204271	−5.3444995589
6	2.3814476377	3.3619628718	−5.0306088531
1	2.3312637079	2.5990368683	−4.2545872235
6	3.5935568411	3.5952698891	−5.6877314150
1	4.4679130093	3.0032148140	−5.4115786471
6	3.6833392707	4.5622799023	−6.6893544242
1	4.6240569351	4.7384274826	−7.2130857433
6	2.5505046785	5.3147672186	−7.0129588432
1	2.5958933731	6.0844974210	−7.7856336668

Table S6: 23 symmetry unique atomic coordinates for DPTZ-DBTO2 molecule in DFT/ωB97X-D/cc-pVDZ C_{2v} GS geometry.

16	−0.000000	0.000000	4.355301
16	−0.000000	6.289294	−1.999404
8	1.273535	−0.000000	5.090434
7	−0.000000	3.885147	−0.171415
6	−0.000000	1.287668	3.099714
6	−0.000000	0.739711	1.814751
6	−0.000000	1.595210	0.718723
1	−0.000000	1.212577	−0.303338
6	−0.000000	2.978019	0.932897
6	−0.000000	3.502288	2.226256
1	−0.000000	4.585127	2.351016
6	−0.000000	2.651586	3.333058
1	−0.000000	3.047568	4.348890
6	1.346974	5.155595	−1.767971
6	1.234267	4.114643	−0.830868
6	2.370637	3.340216	−0.563810
1	2.327894	2.543841	0.177375
6	3.574370	3.588652	−1.221020
1	4.444457	2.975748	−0.980976
6	3.665456	4.594669	−2.176199
1	4.602424	4.782778	−2.701356
6	2.541694	5.370651	−2.451351
1	2.589402	6.172762	−3.189959

Table S7: 23 symmetry unique atomic coordinates for DPTZ-DBTO2 molecule in DFT/M06-2X/cc-pVDZ C_{2v} GS geometry.

16	−0.000000	0.000000	4.369159
16	0.000000	6.218646	−2.077859
8	1.271848	−0.000000	5.104842
7	0.000000	3.892717	−0.149545
6	0.000000	1.287880	3.114666
6	0.000000	0.740426	1.827978
6	0.000000	1.598989	0.733090
1	0.000000	1.221243	−0.291396
6	0.000000	2.981914	0.954070
6	0.000000	3.504619	2.248185
1	0.000000	4.587277	2.371599
6	0.000000	2.650322	3.354163
1	0.000000	3.040401	4.372071
6	1.344536	5.095137	−1.801814
6	1.234725	4.094182	−0.820776
6	2.374601	3.337220	−0.518843
1	2.335527	2.575597	0.257327
6	3.577936	3.562797	−1.186378
1	4.449785	2.965664	−0.918586
6	3.665857	4.526460	−2.184486
1	4.601746	4.695660	−2.716136
6	2.538735	5.285553	−2.492891
1	2.580239	6.056192	−3.263790

Table S8: 65 unique coordinates for DPTZ-DBTO2 molecule in CIS/KTZVP ÇCT geometry.

16	−0.0000020944	0.0387077157	−0.2148450855
16	−0.0000002402	−6.2018816343	−6.6208459633
16	0.0000003831	5.8578082079	−7.1054991544
8	−1.2251604630	0.0117754773	0.5615799354
8	1.2251586717	0.0117647954	0.5615761927
7	−0.0000012880	−3.9311256736	−4.6424132266
7	0.0000001691	3.7035566393	−4.8399818861
6	0.0000034304	−1.2799915883	−1.4214866412
6	−0.0000038559	1.2593772453	−1.4570567259
6	0.0000041439	−0.7790019510	−2.7133246493
6	−0.0000050195	0.6832715727	−2.7555690770
6	0.0000062997	−1.6590963791	−3.7764895061
6	−0.0000063675	1.4706775963	−3.8604863349
1	0.0000022382	−1.3014928430	−4.7882542082
1	−0.0000001521	1.0474734488	−4.8475835165
6	0.0000014115	−3.0165822020	−3.5397202922
6	0.0000011807	2.8727309428	−3.7002847134
6	0.0000012264	−3.5074022349	−2.2412431858
6	−0.0000021723	3.4626092560	−2.3924382614
1	−0.0000013182	−4.5686987009	−2.0828238968
1	−0.0000000642	4.5295306479	−2.2826557387
6	0.0000051925	−2.6335324778	−1.1676284921
6	−0.0000039560	2.6472846048	−1.2960415236
1	−0.0000028656	−3.0041886562	−0.1602902975
1	0.0000029252	3.0786557757	−0.3121324295
6	−1.3435100821	−5.0824865107	−6.3188636358
6	1.3435183610	−5.0825023617	−6.3188442999
6	−1.3509726766	4.9695637544	−6.4585850740
6	1.3509731513	4.9695642494	−6.4585840148
6	−1.2274502251	−4.1100820790	−5.3292945066
6	1.2274358869	−4.1100473801	−5.3293267337
6	−1.2209721432	4.0544159871	−5.3989167271
6	1.2209726697	4.0544128963	−5.3989187017
6	−2.3571063142	−3.3558920438	−5.0298459477
6	2.3571167393	−3.3559170219	−5.0298212260
6	−2.3950635536	3.4342016054	−4.9226116896
6	2.3950653196	3.4342048108	−4.9226078845
1	−2.3268862213	−2.6234438202	−4.2521218622
1	2.3268895975	−2.6234414931	−4.2521232034
1	−2.3236968185	2.7420284198	−4.1140254635
1	2.3236961184	2.7420257883	−4.1140265103
6	−3.5426799964	−3.5462460194	−5.7139119519
6	3.5426733021	−3.5462248575	−5.7139322209

Continued on next page

6	−3.6060677349	3.6904187292	−5.4960463959
6	3.6060675190	3.6904197263	−5.4960455432
1	−4.3972263820	−2.9532704615	−5.4449733804
1	4.3972290402	−2.9532708085	−5.4449736344
1	−4.4789792378	3.1856373009	−5.1297421936
1	4.4789800848	3.1856369553	−5.1297437941
6	−3.6331131939	−4.4807328054	−6.7225796522
6	3.6331213892	−4.4807562228	−6.7225587709
6	−3.7137548343	4.5884536427	−6.5579791721
6	3.7137573021	4.5884564563	−6.5579766905
1	−4.5498493337	−4.6222938528	−7.2630262239
1	4.5498495642	−4.6222957336	−7.2630257595
1	−4.6672554363	4.7821779985	−7.0108271871
1	4.6672548671	4.7821774993	−7.0108284829
6	−2.5208148065	−5.2411683712	−7.0234743975
6	2.5207999496	−5.2411356316	−7.0235043229
6	−2.5944893649	5.2260488404	−7.0231097828
6	2.5944879720	5.2260428584	−7.0231148462
1	−2.5657673127	−5.9804401665	−7.8013137019
1	2.5657683890	−5.9804470795	−7.8013051826
1	−2.6661594256	5.9269877402	−7.8335474755
1	2.6661604541	5.9269898760	−7.8335450788

Table S9: 65 unique coordinates for DPTZ-DBTO2 molecule in TDDFT/B3LYP/cc-pVDZ C_1 CT geometry.

16	0.0000001629	−0.0091354459	−0.2002521986
16	0.0000129585	−6.2949968408	−6.7244210072
16	0.0000022105	6.4422386637	−6.4342779508
8	−1.2557391490	−0.0292734017	0.5833998731
8	1.2557391266	−0.0292728273	0.5834004166
7	0.0000009326	−3.8280598779	−4.8256136540
7	0.0000000868	3.9334962159	−4.7124172549
6	−0.0000028145	−1.2741964338	−1.4593605667
6	0.0000010757	1.2829041244	−1.4268961923
6	0.0000082401	−0.6840734872	−2.7745401154
6	0.0000016751	0.7388726400	−2.7526817862
6	−0.0000121634	−1.5607070381	−3.8819050415
6	−0.0000004891	1.6450367598	−3.8367157539
1	−0.0000275047	−1.1669748153	−4.8950748138
1	−0.0000039845	1.2797030865	−4.8602129680
6	0.0000043093	−2.9228265280	−3.6649699378
6	−0.0000000171	3.0092731183	−3.6021336156
6	0.0000059621	−3.5084727155	−2.3683746443
6	0.0000031376	3.5317543895	−2.2824346154
1	−0.0000080504	−4.5847656859	−2.2426537390
1	−0.0000007297	4.6052109261	−2.1378346150
6	0.0000160039	−2.6472444891	−1.2660123956
6	−0.0000027878	2.6539522372	−1.1967916674
1	−0.0000026209	−3.0581967284	−0.2588753655
1	0.0000004493	3.0412078225	−0.1802148121
6	−1.3504789707	−5.2735091426	−6.2989220344
6	1.3502035825	−5.2729987198	−6.2997069318
6	−1.3549488485	5.3034183359	−6.2393429283
6	1.3549080175	5.3033408297	−6.2394603138
6	−1.2236288967	−4.1967451827	−5.3754889799
6	1.2238394129	−4.1972885742	−5.3748576393
6	−1.2350491133	4.2132571822	−5.3521990039
6	1.2350742262	4.2133247948	−5.3521218674
6	−2.3961133907	−3.4661805518	−5.0572757237
6	2.3957408692	−3.4654198138	−5.0583290216
6	−2.3810533064	3.4315240364	−5.1181378059
6	2.3810082515	3.4314328058	−5.1182647054
1	−2.3258950874	−2.6418074486	−4.3628086988
1	2.3259175936	−2.6419198070	−4.3627212480
1	−2.3274932441	2.6001904227	−4.4269740262
1	2.3274942137	2.6002015430	−4.4269676024
6	−3.6010563649	−3.7690306051	−5.6607497302

Continued on next page

6	3.6015813589	−3.7703006182	−5.6591272873
6	−3.5900216100	3.7214313766	−5.7519866989
6	3.5900865014	3.7215906293	−5.7517831323
1	−4.4703725828	−3.1604307375	−5.4326104103
1	4.4702563666	−3.1602003826	−5.4328916704
1	−4.4584004174	3.1043861386	−5.5329879145
1	4.4583885744	3.1043639386	−5.5330150486
6	−3.7113321700	−4.8336328897	−6.5757696438
6	3.7107961677	−4.8323573098	−6.5773695760
6	−3.6885372865	4.7820593853	−6.6501446499
6	3.6884641170	4.7818834818	−6.6503654525
1	−4.6609428450	−5.0538588357	−7.0516863127
1	4.6610538466	−5.0540401006	−7.0514143106
1	−4.6262555643	5.0036671533	−7.1519206405
1	4.6262686684	5.0036860993	−7.1518910436
6	−2.5918458291	−5.5888493551	−6.8768119896
6	2.5923128231	−5.5897932492	−6.8756503710
6	−2.5575549249	5.5614702134	−6.8997818449
6	2.5576267085	5.5616186488	−6.8995988412
1	−2.6539821852	−6.4177630701	−7.5763278300
1	2.6539506919	−6.4176637345	−7.5765000606
1	−2.6067457375	6.3977541030	−7.5918423357
1	2.6067403636	6.3977353434	−7.5918722521

Table S10: 65 unique coordinates for DPTZ-DBTO2 molecule in ωB97X-D/cc-pVDZ Ç CT geometry.

16	−0.000096000	−0.048617000	4.284276000
16	−0.000187000	−5.446592000	−2.969353000
16	0.000074000	6.359875000	−1.988332000
8	−1.263768000	−0.037967000	5.051957000
8	1.263493000	−0.037928000	5.052092000
7	0.000010000	−3.845747000	−0.282291000
7	0.000051000	3.928167000	−0.166198000
6	−0.000015000	−1.309967000	3.035573000
6	−0.000055000	1.246523000	3.045815000
6	0.000052000	−0.725507000	1.713213000
6	0.000036000	0.715336000	1.737079000
6	0.000090000	−1.595685000	0.624440000
6	0.000072000	1.624177000	0.666036000
1	0.000121000	−1.217619000	−0.400267000
1	0.000135000	1.277567000	−0.369093000
6	0.000035000	−2.961575000	0.854331000
6	0.000015000	2.991202000	0.922252000
6	−0.000041000	−3.547602000	2.157607000
6	−0.000082000	3.493834000	2.230479000
1	−0.000098000	−4.627875000	2.285321000
1	−0.000126000	4.572331000	2.384103000
6	−0.000036000	−2.666881000	3.252757000
6	−0.000119000	2.606151000	3.306490000
1	−0.000105000	−3.059208000	4.271622000
1	−0.000191000	2.970878000	4.334652000
6	−1.360588000	−4.961894000	−2.001797000
6	1.360352000	−4.961999000	−2.001934000
6	−1.348608000	5.231461000	−1.742174000
6	1.348827000	5.231593000	−1.741963000
6	−1.229850000	−4.240029000	−0.789059000
6	1.229807000	−4.240118000	−0.789176000
6	−1.232879000	4.187906000	−0.806412000
6	1.233051000	4.188023000	−0.806225000
6	−2.410156000	−3.889265000	−0.089378000
6	2.410243000	−3.889442000	−0.089650000
6	−2.380080000	3.433018000	−0.522335000
6	2.380284000	3.433259000	−0.521952000
1	−2.319558000	−3.346078000	0.847507000
1	2.319836000	−3.346253000	0.847247000
1	−2.333053000	2.638260000	0.219887000
1	2.333216000	2.638502000	0.220269000
6	−3.649126000	−4.236020000	−0.583117000

Continued on next page

6	3.649124000	−4.236302000	−0.583540000
6	−3.589047000	3.699086000	−1.161721000
6	3.589331000	3.699456000	−1.161133000
1	−4.541557000	−3.958774000	−0.022603000
1	4.541647000	−3.959128000	−0.023137000
1	−4.464763000	3.100778000	−0.904547000
1	4.465069000	3.101245000	−0.903807000
6	−3.767255000	−4.936246000	−1.796089000
6	3.767049000	−4.936547000	−1.796517000
6	−3.681172000	4.703985000	−2.118474000
6	3.681510000	4.704365000	−2.117871000
1	−4.750430000	−5.199589000	−2.186464000
1	4.750154000	−5.199976000	−2.187009000
1	−4.623232000	4.906534000	−2.629388000
1	4.623636000	4.907017000	−2.628623000
6	−2.632713000	−5.294891000	−2.496044000
6	2.632393000	−5.295101000	−2.496331000
6	−2.549695000	5.462826000	−2.409033000
6	2.550002000	5.463086000	−2.408620000
1	−2.711411000	−5.839555000	−3.438250000
1	2.710932000	−5.839775000	−3.438544000
1	−2.595910000	6.267356000	−3.145448000
1	2.596257000	6.267626000	−3.145022000

Table S11: 65 unique coordinates for DPTZ-DBTO2 molecule in M06-2X/cc-pVDZ Ç CT geometry.

16	0.058390	4.279621	−0.000349
16	5.288388	−2.980398	0.000477
16	−6.281771	−1.969032	0.000317
8	0.059638	5.015727	1.231421
8	0.059530	5.015554	−1.232223
7	3.848437	−0.247403	−0.000188
7	−3.874178	−0.154484	0.000083
6	1.320639	3.045022	−0.000316
6	−1.227898	3.063077	−0.000205
6	0.752592	1.747964	−0.000187
6	−0.699207	1.768184	−0.000140
6	1.608774	0.656991	−0.000132
6	−1.583571	0.695056	−0.000039
1	1.235446	−0.359572	−0.000024
1	−1.227275	−0.327576	0.000013
6	2.970315	0.884101	−0.000269
6	−2.949156	0.935686	−0.000005
6	3.533246	2.165289	−0.000358
6	−3.452738	2.232463	−0.000058
1	4.606767	2.286102	−0.000420
1	−4.524113	2.378352	−0.000028
6	2.669915	3.267086	−0.000395
6	−2.581690	3.313439	−0.000164
1	3.064922	4.275000	−0.000485
1	−2.956848	4.328654	−0.000218
6	4.858339	−2.027756	1.346461
6	4.858330	−2.028417	−1.345984
6	−5.167822	−1.725806	1.339850
6	−5.168048	−1.725851	−1.339414
6	4.203023	−0.768345	1.227746
6	4.202989	−0.768934	−1.227919
6	−4.125471	−0.799606	1.228610
6	−4.125675	−0.799649	−1.228382
6	3.881195	−0.078300	2.415765
6	3.881244	−0.079522	−2.416357
6	−3.369263	−0.524633	2.367189
6	−3.369678	−0.524698	−2.367108
1	3.386456	0.878205	2.348023
1	3.386549	0.877035	−2.349213
1	−2.575820	0.206437	2.324104
1	−2.576239	0.206384	−2.324189
6	4.186529	−0.600146	3.641838

Continued on next page

6	4.186619	−0.602026	−3.642141
6	−3.633938	−1.162574	3.568971
6	−3.634570	−1.162669	−3.568826
1	3.933122	−0.042464	4.532746
1	3.933266	−0.044809	−4.533355
1	−3.036386	−0.915854	4.437118
1	−3.037180	−0.915962	−4.437089
6	4.821475	−1.856338	3.751089
6	4.821542	−1.858284	−3.750708
6	−4.639874	−2.107119	3.658952
6	−4.640516	−2.107221	−3.658602
1	5.050035	−2.268167	4.724527
1	5.050135	−2.270632	−4.723918
1	−4.841230	−2.615544	4.592311
1	−4.842041	−2.615668	−4.591912
6	5.145388	−2.551499	2.622877
6	5.145411	−2.552837	−2.622117
6	−5.397906	−2.389909	2.534241
6	−5.398345	−2.389984	−2.533747
1	5.630125	−3.517636	2.693067
1	5.630155	−3.519007	−2.691782
1	−6.197934	−3.118352	2.581574
1	−6.198383	−3.118426	−2.580920

Table S12: The five lowest singlet vertical excitation energies of each symmetry for DPTZ-DBTO2 in C_{2v} point group geometries. Geometries were obtained using RHF, DFT-B3LYP, DFT-TPSSh, DFT-ωB97X-D(B97), DFT-M06-2X(M06) functionals with cc-pVDZ or KTZVP basis sets. Excited state calculations used cc-pVDZ or def2-TZVP basis sets. Data in columns have a geometry optimization method, geometry optimization basis, excited state method and excited state basis as follows:
[1] B3LYP/cc-pVDZ/*GW*-BSE/def2-TZVP, [2] RHF/KTZVP/*GW*-BSE/def2-TZVP, [3] TPSSh+D3-BJ/cc-pVDZ/*GW*-BSE/def2-TZVP, [4] ωB97X-D/cc-pVDZ/ωB97X-D/def2-TZVP, [5] B3LYP+D3-BJ/cc-pVDZ/ωB97X-D/def2-TZVP, [6] TPSSh+D3-BJ/cc-pVDZ/ωB97X-D/def2-TZVP, [7] M06-2X/cc-pVDZ/M06-2X/def2-TZVP, [8] B3LYP/cc-pVDZ/M06-2X/cc-pVDZ, [9] B3LYP/cc-pVDZ/M06-2X/def2-TZVP, [10] B3LYP+D3-BJ/cc-pVDZ/M06-2X/def2-TZVP, [11] TPSSh+D3-BJ/cc-pVDZ/M06-2X/def2-TZVP.

Symmetry	BSE[1]	BSE[2]	BSE[3]	B97[4]	B97[5]	B97[6]	M06[7]	M06[8]	M06[9]	M06[10]	M06[11]
A_1	4.52	4.53	4.39	4.60	4.56	4.56	4.58	4.66	4.55	4.52	4.53
	4.92	4.96	4.78	5.13	5.09	5.07	5.18	5.17	5.16	5.14	5.13
	5.43	5.44	5.33	5.59	5.58	5.57	5.54	5.61	5.51	5.52	5.51
	5.77	5.77	6.02	5.94	5.94	5.92	5.79	5.81	5.75	5.76	5.74
	6.17	6.15	6.23	6.05	6.04	6.03	5.84	5.86	5.80	5.78	5.78
A_2	4.01	4.05	3.87	3.91	3.85	3.86	3.61	3.58	3.55	3.53	3.53
	4.45	4.37	4.17	3.95	3.88	3.89	3.75	3.78	3.73	3.68	3.69
	4.52	4.46	4.26	4.23	4.16	4.16	4.09	4.17	4.10	4.02	4.04
	4.93	4.90	4.74	4.76	4.71	4.72	4.72	4.75	4.69	4.66	4.67
	5.40	5.39	5.32	5.31	5.28	5.29	4.88	4.89	4.85	4.83	4.82
B_1	4.01	4.05	3.87	3.90	3.84	3.84	3.60	3.57	3.54	3.51	3.52
	4.46	4.36	4.16	3.91	3.88	3.89	3.74	3.77	3.72	3.67	3.68
	4.56	4.45	4.26	4.23	4.15	4.16	4.09	4.16	4.10	4.02	4.03
	4.96	4.90	4.73	4.77	4.72	4.72	4.73	4.75	4.69	4.67	4.67
	5.42	5.40	5.33	5.32	5.28	5.29	4.88	4.89	4.84	4.83	4.82
B_2	4.38	4.38	4.22	4.60	4.55	4.56	4.58	4.66	4.54	4.53	4.53
	4.51	4.53	4.38	4.63	4.59	4.57	4.65	4.68	4.61	4.60	4.58
	4.59	4.62	4.44	4.85	4.80	4.78	4.88	4.88	4.84	4.83	4.81
	5.42	5.43	5.33	5.55	5.54	5.53	5.51	5.55	5.47	5.49	5.47
	5.61	5.62	5.45	5.72	5.68	5.67	5.66	5.75	5.67	5.64	5.62

Table S13: The five lowest triplet vertical excitation energies of each symmetry for DPTZ-DBTO2 in C_{2v} point group geometries. Geometries were obtained using RHF, DFT-B3LYP, DFT-TPSSh, DFT-ωB97X-D(B97), DFT-M06-2X(M06) functionals with cc-pVDZ or KTZVP basis sets. Excited state calculations used cc-pVDZ or def2-TZVP basis sets. Data in columns have a geometry optimization method, geometry optimization basis, excited state method and excited state basis as follows:
[1] B3LYP/cc-pVDZ/*GW*-BSE/def2-TZVP, [2] RHF/KTZVP/*GW*-BSE/def2-TZVP, [3] TPSSh+D3-BJ/cc-pVDZ/*GW*-BSE/def2-TZVP, [4] ωB97X-D/cc-pVDZ/ωB97X-D/def2-TZVP, [5] B3LYP+D3-BJ/cc-pVDZ/ωB97X-D/def2-TZVP, [6] TPSSh+D3-BJ/cc-pVDZ/ωB97X-D/def2-TZVP, [7] M06-2X/cc-pVDZ/M06-2X/def2-TZVP, [8] B3LYP/cc-pVDZ/M06-2X/cc-pVDZ, [9] B3LYP/cc-pVDZ/M06-2X/def2-TZVP, [10] B3LYP+D3-BJ/cc-pVDZ/M06-2X/def2-TZVP, [11] TPSSh+D3-BJ/cc-pVDZ/M06-2X/def2-TZVP.

Symmetry	BSE[1]	BSE[2]	BSE[3]	B97[4]	B97[5]	B97[6]	M06[7]	M06[8]	M06[9]	M06[10]	M06[11]
A_1	3.00	3.13	2.98	3.80	3.75	3.75	3.89	3.95	3.87	3.83	3.83
	3.16	3.29	3.13	4.02	3.98	3.98	4.22	4.22	4.18	4.18	4.18
	3.44	3.57	3.42	4.11	4.07	4.06	4.39	4.40	4.38	4.36	4.34
	3.88	4.00	3.85	4.50	4.47	4.46	4.62	4.67	4.61	4.59	4.59
	3.89	4.02	3.87		4.54	4.53		4.64	4.70	4.68	4.67
A_2	2.71	2.86	2.68	3.38	3.30	3.31	3.46	3.47	3.43	3.37	3.39
	3.51	3.63	3.46	3.89	3.83	3.83	3.60	3.58	3.55	3.52	3.53
	3.75	3.87	3.72	3.94	3.86	3.86	3.79	3.88	3.81	3.73	3.73
	4.17	4.30	4.15	4.26	4.21	4.22	4.21	4.20	4.16	4.15	4.21
	4.24	4.39	4.22	4.44	4.40	4.40	4.57	4.62	4.57	4.53	4.53
B_1	2.71	2.86	2.68	3.38	3.30	3.31	3.46	3.47	3.43	3.37	3.38
	3.51	3.63	3.46	3.88	3.82	3.82	3.59	3.56	3.54	3.51	3.51
	3.75	3.87	3.72	3.93	3.85	3.85	3.78	3.87	3.81	3.72	3.72
	4.16	4.31	4.14	4.25	4.21	4.21	4.21	4.19	4.16	4.15	4.15
	4.24	4.39	4.21	4.44	4.40	4.40	4.57	4.62	4.57	4.53	4.53
B_2	2.50	2.67	2.48	3.39	3.33	3.31	3.63	3.59	3.57	3.57	3.55
	3.00	3.13	2.98	3.80	3.75	3.75	3.89	3.95	3.87	3.83	3.83
	3.44	3.57	3.42	4.02	3.98	3.98	4.20	4.22	4.17	4.16	4.15
	3.50	3.61	3.48	4.14	4.10	4.09	4.22	4.24	4.18	4.19	4.18
	3.61	3.72	3.57	4.33	4.30	4.28	4.47	4.51	4.46	4.44	4.43

References

(1) Brémond, E.; Savarese, M.; Adamo, C.; Jacquemin, D. *J. Chem. Theor. Comput.* **2018**, *14*, 3715.
